# Heteromeric assembly of P2X subunits

**DOI:** 10.3389/fncel.2013.00250

**Published:** 2013-12-18

**Authors:** Anika Saul, Ralf Hausmann, Achim Kless, Annette Nicke

**Affiliations:** ^1^Department of Molecular Biology of Neuronal Signals, Max Planck Institute for Experimental MedicineGöttingen, Germany; ^2^Molecular Pharmacology, RWTH Aachen UniversityAachen, Germany; ^3^Department of Discovery Informatics, Grünenthal GmbH, Global Drug DiscoveryAachen, Germany

**Keywords:** P2XR, subunit interface, homomer, heteromer, clustering, ligand binding site

## Abstract

Transcripts and/or proteins of P2X receptor (P2XR) subunits have been found in virtually all mammalian tissues. Generally more than one of the seven known P2X subunits have been identified in a given cell type. Six of the seven cloned P2X subunits can efficiently form functional homotrimeric ion channels in recombinant expression systems. This is in contrast to other ligand-gated ion channel families, such as the Cys-loop or glutamate receptors, where homomeric assemblies seem to represent the exception rather than the rule. P2XR mediated responses recorded from native tissues rarely match exactly the biophysical and pharmacological properties of heterologously expressed homomeric P2XRs. Heterotrimerization of P2X subunits is likely to account for this observed diversity. While the existence of heterotrimeric P2X2/3Rs and their role in physiological processes is well established, the composition of most other P2XR heteromers and/or the interplay between distinct trimeric receptor complexes in native tissues is not clear. After a description of P2XR assembly and the structure of the intersubunit ATP-binding site, this review summarizes the distribution of P2XR subunits in selected mammalian cell types and the biochemically and/or functionally characterized heteromeric P2XRs that have been observed upon heterologous co-expression of P2XR subunits. We further provide examples where the postulated heteromeric P2XRs have been suggested to occur in native tissues and an overview of the currently available pharmacological tools that have been used to discriminate between homo- and heteromeric P2XRs.

## Assembly of P2XRs

### Trimeric structure of P2XRs

Early electrophysiological measurements in bullfrog sensory neurons and single channel analysis of HEK cell-expressed P2X2Rs predicted that there are at least three ATP molecules needed to open a P2X channel (Bean, 1990; Ding and Sachs, 1999). Cross-linking studies and blue-native PAGE analysis of P2X1 and P2X3 receptors heterologously expressed in *Xenopus laevis* oocytes revealed the first biochemical evidence for a trimeric quaternary structure of P2XR channels (Nicke et al., 1998). This rather unexpected architecture was subsequently confirmed by atomic force microscopy (AFM) (Barrera et al., 2005), electron microscopy (EM), single particle analysis (Mio et al., 2005; Young et al., 2008) and finally the first crystal structure of a P2XR, the truncated zebrafish zP2X4R (Kawate et al., 2009), which constituted a major breakthrough in P2XR research. Unexpectedly, the crystal structure of the acid sensing ion channel (ASIC), a member of the ENaC/DEG (epithelial sodium channels/degenerin) superfamily, which shares the same topology and was published around the same time by the Gouaux group, also revealed a trimeric structure, although the two channels show no significant amino acid sequence relationships or similarities in the folding of their extracellular domains (Jasti et al., 2007; Gonzales et al., 2009; Kawate et al., 2009).

The overlapping expression patterns of various P2X subunits, the poor expression of functional P2X_5_ and P2X_6_ homomers in heterologous systems (see below), and the lack of correlation of the functional properties of heterologously expressed homomeric P2X3Rs with P2XRs found in dorsal root ganglions (Lewis et al., 1995), have early led to the assumption that P2XRs, like most ionic receptors, form heteromers. The existence of P2X heteromers is now firmly established but their specific composition and presence in native tissues remains in most cases enigmatic (see chapter Distribution of P2XR subunits). Likewise, their stoichiometry and determinants for subtype specific assembly are largely unclear. Biochemical and/or functional analysis of heterologously expressed P2X2/3 and P2X2/6 heteromers indicates a fixed stoichiometry of P2X2(3)_2_ (Jiang et al., 2003) and P2X(2)_2_6 (Hausmann et al., 2012), respectively. In contrast, a variable, expression-level-dependent stoichiometry for P2X2/6 heteromers was observed in atomic force imaging experiments (Barrera et al., 2007). Co-purification experiments with the P2X1/2 heteromer suggest a P2X1(2)_2_ stoichiometry (Aschrafi et al., 2004). So far, no evidence has been presented for the formation of complexes containing three different subunits.

### Assembly domains and molecular structure of the P2XR

To investigate the role of the transmembrane domains (TMs) in subunit assembly, Torres and colleagues performed co-precipitation studies in HEK cells and found that the association of P2X2 subunits with either itself or P2X3 subunits was prevented if TM2 and the preceding 25 amino acids were deleted (Torres et al., 1999b). To confirm the hypothesis that TM2 rather than the extracellular domain is critical for subunit assembly, these investigators made use of the finding that P2X6 subunits were able to co-immunoprecipitate with P2X1 but not P2X3 subunits (Torres et al., 1999a). Using chimeras in which the extracellular loops between P2X1 and P2X3 subunits were swapped they could demonstrate that only the chimera containing the P2X1 TMs was able to co-immunoprecipitate the P2X6 subunit. In a subsequent study on the hP2X5R splice variant that lacks the C-terminal end of the ectodomain and the outer half of the TM2, it was shown that tethering of the C-terminal end of the ectodomain by membrane insertion of TM2 and the intramembrane positioning of D355 are critical for homotrimeric assembly (Duckwitz et al., 2006). It was concluded that membrane insertion of TM2 restricts the conformational mobility of the ectodomain and thus enables correct positioning of assembly recognition sites located in the ectodomain, while D355 assists in the hydrogen bond-driven transmembrane helix-helix associations. In the zP2X4R crystal structure, however, it is seen that inter-subunit contacts are largely formed between the ectodomains (Kawate et al., 2009). The homotrimeric zP2X4R resembles a chalice, with the large extracellular domain raising ~70 Å above the membrane plane and the six TM helices forming the shape of an hourglass. The single zP2X4 subunit structure has been compared with the shape of a jumping dolphin, in which the two TM helices and the largest part of the extracellular region form the fluke and the upper body, respectively. Attached to the large body domain, a flexible head domain, a dorsal fin, and right and left flippers have been defined. The body domain appears structurally rigid due to extensive β-sheet contacts within a β-sandwich motif. Three interfaces with close contact between adjacent subunits were defined; upper-body-to-upper-body, head-to-body and left-flipper-to-dorsal-fin. Thus, the contacts between neighboring subunits are restricted to the upper ectodomains. The lack of significant contacts between the lower bodies of the extracellular domains enables significant movements in these domains during ATP-induced channel opening (Kawate et al., 2009; Hattori and Gouaux, 2012). It was suggested that the more conserved body domains constitute a common assembly interface in all P2XRs, while the less conserved dorsal fin, head, and left flipper domains guide the subunit-specific assembly by the head-to-body and left-flipper-to-dorsal-fin contacts (Kawate et al., 2009). The low conservation of the latter domains has an important consequence: While homotrimeric P2XRs contain three identical subunit-subunit interfaces, heteromeric P2XRs form always three significantly different interfaces between the “head-to-tail” arranged subunits (see also “Specific characteristics of the ATP sites in heteromeric P2XRs”).

### The intersubunit ATP-binding site

Most of the conserved amino-acid residues involved in the interaction with ATP have been identified in mutagenesis-based studies (Ennion et al., 2000; Jiang et al., 2000; Roberts and Evans, 2004; Yan et al., 2005; Roberts and Evans, 2006; Wilkinson et al., 2006; Young et al., 2006; Fischer et al., 2007; Zemkova et al., 2007; Roberts et al., 2008; Donnelly-Roberts et al., 2009; Evans, 2009; Roberts et al., 2009; Browne et al., 2010; Evans, 2010; Bodnar et al., 2011). Based on disulfide cross-linking experiments, in which some of these residues were substituted by cysteine residues, it was concluded that the agonist binding site is located at the interface between two neighboring subunits (Marquez-Klaka et al., 2007). Crystallization of the zP2X4R showed that these residues surround a large intersubunit cavity, which was proposed to constitute the ATP binding site. This cavity is formed between two complementary half-shells contributed by the adjacent subunits A and B. When viewed from the side (i.e., in parallel to the membrane plane), the upper left and the lower right boundaries of each ATP-binding site are constituted by the upper body and the left flipper of subunit A and the lower body and dorsal fin of subunit B, respectively (Kawate et al., 2009). By labeling of engineered cysteines with thiol-reactive ATP-analogs (Jiang et al., 2011) and by voltage-clamp fluorometry studies (Lörinczi et al., 2012), it was confirmed that this cavity constitutes the ATP binding site and that the flexible head domain of subunit A that projects over the binding site moves substantially during ligand binding and/or channel gating (Hattori and Gouaux, 2012; Jiang et al., 2012; Lörinczi et al., 2012). The zP2X4 structure provided a basis for rational mutant design, *in silico* docking, and molecular dynamics simulations which were crucial to identify the three lateral ion-access-pathways, and to improve our understanding of the molecular mechanisms of ligand binding and channel gating (Jiang et al., 2010; Rokic et al., 2010; Allsopp et al., 2011; Bodnar et al., 2011; Jiang et al., 2011; Kawate et al., 2011; Samways et al., 2011; Wolf et al., 2011; Du et al., 2012a; El-Ajouz et al., 2012; Jiang et al., 2012; Lörinczi et al., 2012; Roberts et al., 2012; Hausmann et al., 2013). However, docking of ATP proved difficult due to the spatial diversity of the ATP-binding pockets of different P2X subtypes, the high flexibility of the critical lysine residues, and a multitude of possible binding-modes within the relatively large (compared to the ATP molecule) binding pocket. The precise mode of ATP-binding was only determined by crystallization of an ATP-bound zP2X4R (Hattori and Gouaux, 2012). The phosphate chain and the adenine ring of the ATP molecule are folded in an U-shaped configuration within the ATP-binding pocket. The phosphate oxygens of ATP are coordinated by the side chains of K70 and K72 within the lower body of subunit B and N296, R298, and K316 within the upper body of subunit A (zP2X4 numbering). In agreement with labeling experiments at the P2X2R using a thiol-reactive ATP-analog (Jiang et al., 2011), the adenine base of ATP is making hydrophobic interactions with L191 of the lower body and I232 of the dorsal fin (subunit B). It is further stabilized by hydrogen bonds with the side chain of T189 and the backbone of K70, both located within the lower body of subunit B. The ribose moiety is facing the solution (Lörinczi et al., 2012) and recognized solely by hydrophobic interactions with L217 within the dorsal fin of subunit B (Hattori and Gouaux, 2012).

### Specific characteristics of the ATP-binding sites in heteromeric P2XRs

**Figure 1:**
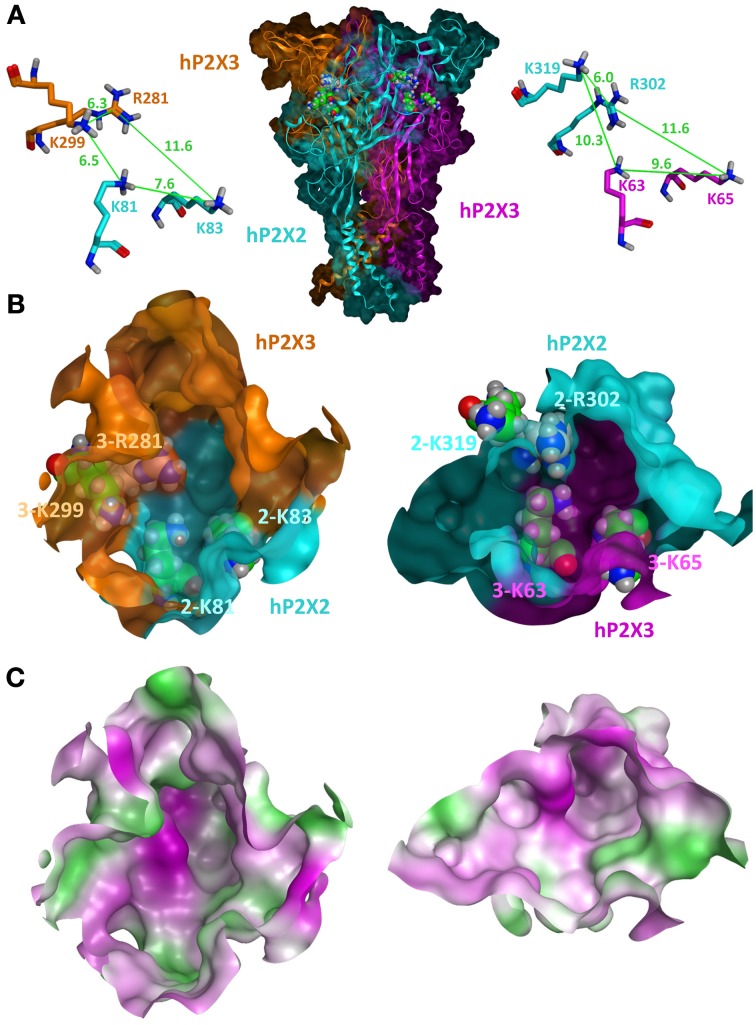
**Homology model of the closed state of the human P2X2/3R. (A)** A side view of the heterotrimeric hP2X2(3)_2_R in which both heteromeric interfaces are visible is shown in the middle. The P2X2 subunit is colored in cyan and the two P2X3 subunits are colored in orange and pink. Selected basic residues important for ATP binding are shown as spheres within the two intersubunit ATP-binding sites. For clarity, the same residues are shown as sticks in a close-up view with depiction of the distances between their side chains. **(B)** Frontal close-up views of the two heteromeric ATP-binding sites with a partial transparent surface to indicate the orientation of the residues. The coloring of the subunits is the same as in **(A)**. Differences in the three-dimensional structures and volumes of the hydrophilic cavities are clearly seen. **(C)** Surface representations of the same view as in **(B)** with gradual depiction of hydrophobic (green) or hydrophilic (pink) areas/residues. Neutral areas are shown gradually white. The hP2X2/3R homology model was generated and visualized by the molecular modeling program MOE2012.10 (Molecular Operating Environment 2012, CCG, Montreal, Canada) using the apo zP2X4 crystal structure (PDB entry 3H9V; (Kawate et al., 2009) as a template as previously described (Wolf et al., 2011; Hausmann et al., 2013).

Under the condition of similar conformations of the subunits and their uniform arrangement (Kawate et al., 2009; Hattori and Gouaux, 2012), three equivalent intersubunit ATP-binding sites can be assumed in one homomeric P2XR. Based on the zP2X4 structure, homology models of the ATP-binding sites of homomeric P2X1, P2X2, P2X3, P2X4, and P2X7 receptors have been generated so far (Keceli and Kubo, 2009; Roger et al., 2010; Allsopp et al., 2011; Bodnar et al., 2011; Jiang et al., 2011; Wolf et al., 2011; El-Ajouz et al., 2012; Jiang et al., 2012; Lörinczi et al., 2012; Roberts et al., 2012; Schwarz et al., 2012; Hausmann et al., 2013). As outlined above, three significantly different intersubunit binding sites are formed in the case of heteromeric P2X assemblies: one between two identical subunits and two at the heteromeric interfaces A/B and B/A. The structural differences of the latter two are mainly due to structural differences in the upper bodies and left flippers as exemplarily shown for P2X2 and P2X3 subunits (Figure 1) and lead to different interfaces and volumes of the hydrophilic cavities. In addition, the overlapping head domains differ significantly in their backbone conformation and side chain orientation. These domains are characterized by a highly conserved pattern of three disulfide bridges that constrain the rather non-conserved sequences between the cysteine residues in loops of different length and structure. Although these domains do not directly contribute to ATP binding, they appear to be important for channel gating and might have crucial influence on antagonist selectivities (Wolf et al., 2011; El-Ajouz et al., 2012; Hattori and Gouaux, 2012; Jiang et al., 2012; Lörinczi et al., 2012; Roberts et al., 2012). The side chains of residues responsible for the coordination of ATP, e.g., as illustrated for selected basic residues in Figure 1, are in different spatial orientation and in unequal distances relative to each other. Evidence for varying distances and/or orientation of residues critical for ATP binding was also obtained in cysteine cross-linking experiments at P2X1/P2X2 interfaces in the respective heteromer (Marquez-Klaka et al., 2009). Here, co-expression of K68C-P2X1 and F289C-P2X2 subunits resulted in spontaneous intersubunit cross-linking, while co-expression of F291C-P2X1 and K69C-P2X2 subunits did not produce significant amounts of SDS-resistant dimers. As a consequence of the spatial orientations, unequal binding sites are formed where ligands likely adopt substantially different binding modes and consequently different binding affinities and specificities. This is reflected in distinct pharmacological characteristics of heteromeric and homomeric P2XRs (Gever et al., 2006; Jarvis and Khakh, 2009; Coddou et al., 2011b) and may open the possibility for the development of ligands that are able to specifically block heteromeric P2XRs. However, so far it is not known, if a heteromeric P2XR can be blocked by specific targeting of one of the two heteromeric ligand binding sites or how many binding sites have to be occupied by antagonists to inhibit the channel.

### Cooperativity of ATP binding in P2XRs

Binding of agonist is supposed to induce channel opening by a closing movement of the head domain that is further propagated to the TM domains (Hattori and Gouaux, 2012; Jiang et al., 2012; Lörinczi et al., 2012). Dose response curves for ATP yield generally Hill-slopes greater than one (Bean, 1992; Brake et al., 1994) indicating that more than one agonist molecule binds before channel opening occurs. However, analyses of dose response curves are deceptively complex (Colquhoun, 1998) and the Hill-slope can only serve as a rough estimate of the minimum number of binding sites. The reduced Hill coefficients observed for agonist dose response curves at heteromeric P2XRs (Torres et al., 1998; Haines et al., 1999; Surprenant et al., 2000; Jiang et al., 2003) might suggest that the heteromers require fewer agonist molecules for opening but could also be explained by factors like altered cooperativity or desensitization. From electrophysiological analysis and mathematical modeling of the activation and deactivation kinetics of the P2X2, P2X3, and P2X7 receptors it was concluded that two ATP molecules are sufficient to open the channels while occupation of three binding sites enables dilation of the pore in P2X2 and P2X7 subtypes (Karoly et al., 2008; Yan et al., 2010; Khadra et al., 2012). As detailed below, co-expression of wt (wild type) P2X6 subunits rescued ATP-elicited currents of P2X2 subunits with defective ATP-binding sites. Since receptors with one non-functional ATP-binding site can be activated, this supports the model in which binding of two ATP molecules is sufficient for channel gating (Wilkinson et al., 2006; Hausmann et al., 2012). Similar studies with concatenated subunits indicate that this is also valid for homomeric P2X2Rs (Stelmashenko et al., 2012). In contrast, photoaffinity labeling data at a homomeric non-desensitizing P2X2/1 chimera are better described by a model in which three BzATP molecules must bind to open the channel (Bhargava et al., 2012). In case that binding of two ATP molecules is sufficient to induce channel opening, ATP would need to bind and induce closure of the head domain in at least two of the three distinct ATP-binding sites in heteromeric P2XRs. For antagonists, the situation is likely different. Like in other ligand-gated ion channels, orthosteric antagonists of P2XR are generally larger molecules and supposed to block the closing movement or even induce an opening of the ATP-binding pocket (Du et al., 2012b), thereby preventing the initial gating step. Provided that simultaneous symmetric conformational changes of the three subunits should be favored, it might depend on the size of antagonist if targeting of one ATP binding sites is sufficient to prevent the ATP-induced gating of the whole trimeric channel. In case of the muscle-type nAChR for example, it appears, that occupation of only one agonist binding site by specific peptide antagonists is sufficient to inhibit channel opening, although it has to be considered that this channel has only two agonist binding sites (Groebe et al., 1995).

## Properties of heterologously expressed P2XRs

### Homomeric expression

When heterologously expressed, most mammalian P2X subunits readily form homotrimeric complexes with functional and pharmacological properties described below. For P2X5Rs species-specific differences in expression efficiency and functional properties such as ion permeability were observed (Collo et al., 1996; Garcia-Guzman et al., 1996; Cox et al., 2001; Jensik et al., 2001; Ruppelt et al., 2001; Diaz-Hernandez et al., 2002; Wildman et al., 2002). This subunit occurs in humans predominantly as a non-functional splice variant (Le et al., 1997; Bo et al., 2003; Duckwitz et al., 2006) and expression of the mouse and rat isoforms is inefficient (Collo et al., 1996; Cox et al., 2001; Wildman et al., 2002). For P2X6Rs, heteromerization with other subunits appears to be obligatory for correct folding and trimeric assembly (see below) (Collo et al., 1996; Soto et al., 1996b; Nawa et al., 1998; King et al., 2000; Aschrafi et al., 2004; Barrera et al., 2005).

P2XRs are generally classified in rapidly desensitizing (P2X1 and P2X3), and slowly or non-desensitizing (P2X2, P2X4, P2X5, and P2X7) receptors although desensitization properties can change under cell-free conditions (Ding and Sachs, 2000). For P2X2, P2X4, and P2X7 receptors, additional permeability states have been described (Khakh et al., 1999; Virginio et al., 1999a,b; Khadra et al., 2012, 2013), for details see (Kaczmarek-Hajek et al., 2012), which are little understood on the molecular level.

### Agonist pharmacology of homomeric P2XRs

As a recent and detailed review of P2XR pharmacology is available (Coddou et al., 2011b), the following paragraphs summarize only some of the key functional properties of homomeric P2XRs with a focus on those characteristics that have been used to differentiate homomeric and heteromeric P2XRs.

The EC_50_ values for ATP range from submicromolar concentrations for P2X1, P2X3, and P2X5 to low micromolar concentrations for P2X2 and P2X4 receptors. At the P2X7R, EC_50_ values for ATP range from about 0.1 to 1 mM for the rat and mouse isoforms, respectively. BzATP is at least one order of magnitude more potent at this receptor (Rassendren et al., 1997) but has also considerable activity at other P2X isoforms (Anderson and Nedergaard, 2006). Agonist potency at P2X7Rs decreases if Ca^2+^ or Mg^2+^ are present in the recording solution. Ca^2+^ decreases the potency of orthosteric agonists independently of the free agonist concentration, thus acting as an allosteric inhibitor (Yan et al., 2011). In the case of Mg^2+^, the inhibition seems to be due to both an inhibitory Mg^2+^ binding site and a lower or absent agonist activity of Mg^2+^-bound ATP at the P2X7R (Virginio et al., 1997; Klapperstück et al., 2001; Acuna-Castillo et al., 2007). Differences in the sensitivity to free ATP and Mg^2+^-bound ATP were also reported for P2X2 and P2X4 receptors but not seen with P2X1, P2X2/3, and P2X3 receptors (Li et al., 2013).

All P2XR agonists known so far are nucleotide analogs. ATPγS and αβ-meATP are metabolically more stable and widely used to investigate ATP-gated channels in native tissues. In addition, 2-MeSATP, diadenosine polyphosphates, and other nucleoside triphosphates are used as P2X agonists. Interestingly, fast desensitization and slow recovery from desensitization of P2X1R and P2X3R seems to be associated with the formation of high-affinity binding sites for ATP, αβ-meATP, and the antagonist trinitrophenyl-ATP (TNP-ATP). At homomeric P2X2Rs, αβ-meATP is an agonist with very low potency (EC_50_ >100 μM), while homomeric P2X4Rs are activated in a species-dependent manner with EC_50_ values ranging from 5 to 100 μM. P2X7Rs are not readily activated by αβ-meATP (EC_50_ >> 100 μM). In heteromeric assemblies, P2X1, P2X3, P2X5, or P2X6 subunits appear to confer αβ-meATP sensitivity. In contrast, the diadenosine tetraphosphate Ap_4_A, is a full and potent agonist at the homomeric P2X2R (Pintor et al., 1996; Wildman et al., 1999a), but is inactive at the heteromeric P2X2/6R (King et al., 2000).

### Antagonist pharmacology of homomeric P2XRs

The large polysulfonated naphtylurea suramin and the pyridoxalphosphate derivative PPADS are among the earliest and still most widely used P2XR antagonists. Suramin also inhibits other purinergic receptors and G-proteins and more specific analogs (“NF compounds”) have been developed. Of these, NF449 is highly selective for P2X1Rs and NF770 is moderately selective for P2X2Rs. PPADS is a more specific inhibitor for P2 receptors but acts in a non-competitive way. More potent analogs (“MRS compounds”) with certain selectivity for P2X1 (MRS2220), P2X1 and P2X3 (MRS2257), and P2X1, P2X3, and P2X7 (MRS2159) have been developed (Coddou et al., 2011b).

The nucleotide analog TNP-ATP is a competitive antagonist with nanomolar affinity for recombinant P2X1, P2X3, and P2X2/3 receptors, and micromolar affinity for recombinant P2X2 and P2X4 receptors (King et al., 1997; Virginio et al., 1998). Another nucleotide-based antagonist diinosine pentaphosphate (IP_5_I) showed improved stability in native tissues and is a potent and selective P2X1R antagonist (King et al., 1999). Oxidized ATP is used as an irreversible P2X7 antagonist but requires extensive pre-incubation. New types of mostly non-nucleotidic and subtype-specific antagonist have been developed during the last two decades. These efforts focused mainly on the P2X3 and P2X7 subtypes that appeared to be the most relevant drug targets. Among others, the P2X3 and P2X2/3-selective antagonists RO-51, RO-3, TC-P262, AF353 (RO-4), A-317491 and the P2X7-selective antagonists A-438079, A-740003, A-804598, A-839977, AZ 11645373, AZ 10606120 are commercially available. In particular for P2X7Rs, however, species specificity has to be considered. The commonly used inhibitors KN-62 and BBG for example, are selective for human and rat P2X7 isoforms, respectively (Donnelly-Roberts et al., 2009).

At the P2X4R, which has been difficult to target, 5-BDBD has been shown to be a comparably potent blocker (IC_50_ ~ 0.5 μM) (Donnelly-Roberts et al., 2008) and very recently, PSB-12054 was introduced as hP2X4R antagonist with submicromolar potency (IC_50_ ~ 0.2 μM) (Hernandez-Olmos et al., 2012). In addition, more specific P2X2-antagonists, the anthrachinone derivatives PSB-10211 and PSB-1011, were introduced (Baqi et al., 2011).

### Allosteric modulators of P2XRs

Acidification enhances responses of agonists and suramin at the rat P2X2R (King et al., 1997). Likewise, P2X2/3 (Stoop et al., 1997) and, with a distinct pattern, P2X2/6 (King et al., 2000) and P2X1/2 (Brown et al., 2002) receptors are modulated by protons (see below). Mutation of H319 to alanine removed the potentiating effect of acidification on rat P2X2Rs (Clyne et al., 2002). Its characteristic modulation by protons can help to distinguish P2X2Rs since all other homomeric P2XRs are inhibited by acidification [for details see (Coddou et al., 2011b)].

Mg^2+^ was recently shown to directly inhibit P2X1, P2X3, and P2X7 receptors, while P2X2 and P2X2/3 receptor responses were insensitive or less sensitive to Mg^2+^ when activated by supermaximal concentrations of free ATP (Li et al., 2013).

The trace metals Zn^2+^ and Cu^2+^ allosterically modulate P2XRs in complex subtype- and species-specific ways and via non-conserved binding sites [for details see (Coddou et al., 2011a)]. In summary, Zn^2+^ inhibits P2X1 and P2X7 receptor currents and modulates P2X2, P2X3, P2X4, and P2X5R currents in a biphasic way, i.e., it potentiates at low concentrations and inhibits at high concentrations. The Zn^2+^ binding site of the rat P2X2R has been localized at the interface between adjacent subunits (Nagaya et al., 2005). Like zinc, copper modulates the rat P2X2R in a biphasic way, while the human P2X2R and the P2X7R are only inhibited by zinc and copper (Tittle and Hume, 2008; Coddou et al., 2011a). A peculiarity of the P2X4R is that it is differentially modulated by Zn^2+^ and Cu^2+^. While Zn^2+^ and Cu^2+^ both coordinate at the inhibitory allosteric site, Zn^2+^ can additionally bind to a positive allosteric site explaining the different modulatory effects of zinc and copper at the P2X4R (Coddou et al., 2003, 2007, 2011a).

The antiparasitic agent ivermectin (IVM) has agonist properties at invertebrate glutamate receptors (GluRs) and is also pharmacologically active at a number of other ligand-gated ion channels. At homomeric and heteromeric P2X4Rs it is an allosteric modulator (Khakh et al., 1999; Priel and Silberberg, 2004). In the absence of efficient P2X4R inhibitors, it has been widely used as a tool to dissect P2X4 subunit containing receptors. However, more recent studies show that IVM can also species-specifically potentiate P2X7R current amplitudes (Casas-Pruneda et al., 2009; Surprenant and North, 2009; Nörenberg et al., 2010).

### Pharmacology of heteromeric receptors

In most functionally characterized heteromers (see below) the kinetic and ligand-binding properties of the constituting subunits are combined and the slowly desensitizing subunit generally dominates the kinetic, while the subunit with higher affinity for agonist and/or antagonist appears to increase the sensitivity of the heteromer for the respective ligand.

In agreement with this, the P2X2/3 heteromer appears to adopt largely the pharmacological profile (agonist potency, TNP-ATP sensitivity) of the P2X3 subunit, which is present twofold within the complex (Jiang et al., 2003). Although their stoichiometry is not known, the P2X1/4R and the P2X1/5R also show a pharmacological profile more similar to the P2X1R and kinetic properties that resemble the P2X4R or P2X5R, respectively (Coddou et al., 2011b). The often slightly lower potency of agonists at the heteromers is too subtle for a true discrimination from the P2X1 or P2X3 homomer and most likely reflects a lower affinity for ATP at the heteromeric ATP-binding sites. In case of the heteromeric P2X2/6 or P2X4/6 receptors the pharmacological properties are similar to that of the P2X2R or P2X4R, respectively (Coddou et al., 2011b). For the P2X2/6R a stoichiometry of two P2X2 and one P2X6 subunits was suggested (Hausmann et al., 2012). The heteromeric P2X1/2R shows a kinetic and pharmacological profile that resembles the P2X1R, although a P2X1(2)_2_ stoichiometry has been suggested (Aschrafi et al., 2004). However, it adopted the pH sensitivity from the P2X2 subtype (Brown et al., 2002). The H^+^ and Zn^2+^ sensitivity of the P2X2 subtype are also conferred to the P2X2/3 and P2X2/6 heteromers (Li et al., 1996; Stoop et al., 1997; King et al., 2000).

There are a few compounds that appear to be able to discriminate between homomeric and heteromeric P2XRs. The photoreactive [γ−^32^P]8-Azido-ATP is an effective agonist at homomeric P2X3Rs but not at heteromeric P2X2/3Rs. It also efficiently labeled homomeric P2X3Rs, but was inefficient at homomeric P2X2Rs and heteromeric P2X2/3Rs (Koshimizu et al., 2002). Likewise, Ip_5_I was shown to inhibit α,βme-ATP-induced responses of homomeric P2X1 and P2X3 receptors with low micromolar potency, but is virtually inactive at the heteromeric P2X2/3R and the homomeric P2X2R (King et al., 1999; Dunn et al., 2000). Also RO-85, an orally bioavailable drug-like P2X3R antagonist, is selective for the P2X3R over the P2X2/3R and other P2XR subtypes (Brotherton-Pleiss et al., 2010). Interestingly, all these substances appear to lose their affinity at the heteromeric receptor despite the fact that at least one P2X3-P2X3 interface is preserved in the heteromer. Possible explanations for this discrepancy would be, that the P2X3-P2X3 interface is markedly altered by inclusion of the single P2X2 subunit in the complex and/or that occupation of one interface is insufficient and more than one ligand has to bind to produce an efficient channel block. So far, no compound has been identified, that is selective for any of the heteromeric receptors. Thus, it remains questionable, if the two requirements for selective heteromer targeting (1) selective binding to one (or both) heteromeric interfaces and (2) efficient blockade of the whole receptor by occupation of one (or both) heteromeric interface(s) can be fulfilled. In case of small antagonists, the specific recognition area (contributed by both subunits) might be too small and the critical lysine residues too flexible to allow high selectivity. Also, the small ligand volume and the comparably few interactions with both subunits might not provide sufficient steric hindrance to allosterically block the gating movements in all three subunits.

In this regard, it is also important to consider that P2XRs differ substantially from other ligand-gated ion channels in having three intersubunit ion access pathways that widen during channel opening (Kawate et al., 2011; Samways et al., 2011; Hattori and Gouaux, 2012; Roberts et al., 2012). In combination with the high flexibility of the lower ectodomains and their linkers to the TMs, this might enable P2XRs to tolerate or compensate also more or less pronounced antagonist induced conformational changes before a complete channel block occurs.

## Distribution of P2XR subunits

Although transcripts for more than one P2X subunit are found in most cell types (Table 1), there is good evidence (in particular from studies on knockout mice) for homomeric P2X1, P2X2, P2X3, P2X4, and P2X7 receptors in at least some native tissues (e.g., Cockayne et al., 2005; Finger et al., 2005; Sim et al., 2007; Nicke, 2008). However, properties of heterologously expressed homomeric P2XRs more often do not match with those observed in native tissues. In addition to the heteromers described below and yet unidentified combinations of two or three different P2X subunits, splice variants and interacting proteins most likely contribute to the diversity of P2XR signaling.

**Table 1:** **Summary of the distribution of P2XR subunits in selected mammalian cell types**.

**Cell line/type**	**Identified transcripts**							**Identified protein**							**Functional data similar to**
	**1**	**2**	**3**	**4**	**5**	**6**	**7**	**1**	**2**	**3**	**4**	**5**	**6**	**7**	
T-cells: Jurkat cell line^(^^1^^)^	(+)			+	+		(+)	+			+			+	Ca^2+^ imaging, siRNA: P2X1, P2X4, P2X7
T-cells: human primary CD4+^(^^1^^)^	(+)			+	+		+	+			+				Pharmacological inhibition, Ca^2+^ imaging: P2X1, P2X4
Mast cells: LAD2 and human lung mast cells^(^^2^^)^	+			+		(+)	+								Patch clamp: P2X1, P2X4, P2X7
Freshly isolated mouse peritoneal macrophages^(^^3, 4, 46^^)^	(+)			+			+	+			+				KO mouse: P2X1, P2X4
J774 cells^(^^5^^)^			+	+	(+)	+	+		+		+			+	Ca^2+^ imaging, patch clamp: P2X4, P2X7
Mouse spleen macrophages^(^^5^^)^			+	+	+	+	+	(+)	(+)	+	+	+	+	+	
Human B lymphocytes^(^^6, 7^^)^	+			+			+	+	+		+			+	
HeLa cells^(^^8^^)^			(+)	+	+	+	+							+	Ca^2+^ imaging, changes in ATP-induced apoptosis: P2X7
Myocytes from renal artery^(^^9^^)^	+			+											Patch clamp: P2X1, P2X1/4
Endothelial cells^(^^10, 11, 12^^)^	+			+	(+)		+				+			+	Ca^2+^ imaging: P2X4; KO mouse: P2X4, P2X1; patch-clamp: P2X7
Dorsal root ganglia (DRG) neurons^(^^13, 14, 15, 16, 17^^)^		+ ^a^	+	+					+	+		+			Patch clamp: P2X3, P2X2/3; KO mouse: P2X3, P2X2, P2X2/3
Nodose ganglia neurons^(^^13, 15^^)^		+	+	+					+	+					Patch clamp, KO mouse: P2X2, P2X2/3
Superior cervical ganglion (SCG) neurons^(^^13, 18, 19^^)^	(+)	+	+	+	+	+	(+)	+	+	(+)	+		+		Patch clamp, Ca^2+^ imaging: predominantly P2X2; KO mouse: P2X1
Urinary bladder afferent neurons^(^^15, 20, 21, 22^^)^									+	+					Bladder function, patch clamp: P2X3, P2X2/3; KO mouse: P2X2, P2X3, P2X2/3
P19 cells^(^^23, 24, 25, 26^^)^															Ca^2+^ imaging: P2X2, P2X2/6, P2X4; RNAi: P2X2, P2X7
Undifferentiated		(+)	+	+	+	(+)	(+)		(+)	(+)	+		(+)		
Differentiated to progenitors	(+)	+	(+)	(+)	+	+	+	(+)	+	(+)	(+)		+	(+)	
Neuronally differentiated neural progenitors^(^^27^^)^		+	(+)	(+)	(+)	+									
Astrocytes^(^^28, 29, 30, 31^^)^	+	(+)	+	+	+	+	+	+	+	+	+		+	+	Patch clamp: P2X1/5; YO-PRO uptake, RNAi: P2X7
Microglia^(^^32, 33, 34, 34, 35, 36, 46^^)^															Nerve injury, KO mouse: P2X4; patch clamp: P2X4, P2X7; YO-PRO uptake: P2X7
Freshly isolated	(+)			+			+								
Primary culture											+			+	
BV-2 cells				+		+	+				+				
C8-B4 cells			(+)	+			+				+			+	
Oligodendrocytes^(^^38^^)^														+	Ca^2+^ imaging: P2X7
Oligodendrocytes (progenitors)^(^^37^^)^								+	+	+	+			+	Ca^2+^ imaging, patch clamp: P2X7
Human salivary gland epithelial (HSG) cells^(^^39^^)^								+	+	+	+	+	+		
Human embryonic kidney (Hek) 293 cells^(^^39^^)^															
Normal conditions											(+)	(+)			
Grown past confluence								+		+	+	+	+		
Vascular smooth muscle cells^(^^40, 41, 42^^)^	+	+		+				+ ^b^		+ ^c^	+	+		+	Patch clamp: P2X1^b^
Non-cystic fibrosis epithelial cells (16HBE14o^−^)^(^^43^^)^						+						+	+		
Cystic fibrosis cells (IB3-1)^(^^43^^)^				+	+	+					+	+	+		RNAi, Ca^2+^ imaging: P2X4, P2X6
Adrenal gland pheochromocytoma (PC12) cells^(^^44, 45^^)^															Patch clamp, Ca^2+^ imaging: P2X2
Undifferentiated		+							+						
NGF-differentiated	+	+	+	+	+	+	+	+	+	+	+	+	+	+	

a Except primates,

b except septal vessels,

c except cerebral vessels.

References in table:

1 Woehrle et al., 2010a;

2 Wareham et al., 2009;

3 Sim et al., 2007;

4 Ulmann et al., 2010;

5 Coutinho-Silva et al., 2005;

6 Sluyter et al., 2001;

7 Wang et al., 2004;

8 Welter-Stahl et al., 2009;

9 Harhun et al., 2010;

10 Yamamoto et al., 2000;

11 Harrington et al., 2007;

12 Wilson et al., 2007;

13 Lewis et al., 1995;

14 Cockayne et al., 2000;

15 Cockayne et al., 2005;

16 Compan et al., 2012;

17 Serrano et al., 2012;

18 Xiang et al., 1998,

19 Calvert and Evans, 2004;

20 Vlaskovska et al., 2001;

21 Zhong et al., 2001;

22 Zhong et al., 2003;

23 Da Silva et al., 2007;

24 Resende et al., 2007;

25 Resende et al., 2008;

26 Yuahasi et al., 2012;

27 Schwindt et al., 2011;

28 Franke et al., 2001;

29 Kukley et al., 2001;

30 Lalo et al., 2008;

31 Yamamoto et al., 2013;

32 Tsuda et al., 2003;

33 Qureshi et al., 2007,

34 Raouf et al., 2007;

35 Ulmann et al., 2008;

36 Toulme et al., 2010;

37 Agresti et al., 2005;

38 Matute et al., 2007;

39 Worthington et al., 1999;

40 Nori et al., 1998;

41 Lewis and Evans, 2000;

42 Lewis and Evans, 2001;

43 Liang et al., 2005;

44 Michel et al., 1996;

45 Sun et al., 2007;

46 Hickman et al., 2013.

## Heterooligomerization of P2XR subunits

In a systematic biochemical analysis of pairwise co-expressed Flag- and HA-tagged rat P2X subunits in HEK cells (Torres et al., 1999a) it was found that (a) all subunits, except for P2X6 subunits, were able to homo-oligomerize (see also Aschrafi et al., 2004; Barrera et al., 2005), (b) P2X7 subunits did not hetero-oligomerize with other subunits (see also Nicke, 2008; Boumechache et al., 2009), and (c) the following pairs could be mutually co-purified: P2X1/2^*^, P2X1/3, P2X1/5^*^, P2X1/6, P2X2/3^*^, P2X2/5^*^, P2X2/6^*^, P2X3/5, P2X4/5, P2X4/6^*^, and P2X5/6. Heteromerization between P2X4 and P2X5 subunits was recently confirmed in an ELISA assay where a strong increase in the surface expression of a trafficking deficient P2X5 mutant by co-expression of the P2X4 subunit was observed (Compan et al., 2012). However, this heteromer has not been further characterized so far. In addition, P2X1/4^*^ and P2X4/7^*^ pairs were identified in co-purification studies (Nicke et al., 2005; Guo et al., 2007). Pairs marked with ^*^ have also been functionally investigated and partly confirmed in additional studies that are described below. It has to be noted, however, that the differentiation of a heterotrimeric assembly between two given subunits or association of their respective homotrimeric complexes can generally not be achieved by standard co-purification protocols. In particular, in case of the P2X4/P2X7 interaction, data from more detailed biochemical analysis are in favor of the latter (Nicke, 2008; Antonio et al., 2011).

### P2X1/2 heteromers

#### Biochemical evidence

Biochemical evidence for heteromeric P2X1/2Rs was further confirmed by studies in *Xenopus laevis* oocytes. In blue-native (BN)-PAGE and disulfide cross-linking studies, the metabolically labeled heterotrimeric P2X1/2R complexes were directly visualized and discriminated by their size from the respective homomers (Aschrafi et al., 2004; Marquez-Klaka et al., 2009). These data clearly excluded the possibility that the co-purification experiments were biased by artificial aggregation of the overexpressed protein or clustering of homotrimeric receptors (see below). Interestingly, no homotrimeric His-P2X1 complexes were detected in the BN-PAGE study and functional analysis of the receptors formed from substituted cysteine mutant P2X1 and P2X2 subunits revealed no current corresponding to homomeric P2X1Rs unless this subunit was injected in more than 6-fold excess. This suggests that in the presence of P2X2 subunits, the P2X1 subunit assembles preferentially as heteromer. However, discrepancies in the amount and speed in which the subunits are expressed could also account for this observation. Further analysis of the selectively radioiodinated heteromers in the plasma membrane revealed significantly more radioactivity in the band corresponding to the P2X_2_ subunit than in the His-tagged P2X_1_ subunit, suggesting a P2X1(2)_2_ stoichiometry (Aschrafi et al., 2004).

#### Functional evidence

In a carefully conducted study by Brown et al. (2002) the subtle differences between the fast desensitizing homomeric P2X1R and the heteromeric P2X1/2R could be discriminated by their sensitivity to extracellular pH. In contrast to P2X1 homomers, which show decreased agonist potency at acidic pH and are not affected by alkaline pH, agonist efficacy at the P2X1/2 heteromer is increased under both alkaline and acidic pH (Brown et al., 2002). In contrast to the biochemical studies, hetero-oligomerization was found to be inefficient in this study and only one in six oocytes showed the pH-potentiated P2X1-like responses. The difficulty to resolve the P2X1/2 heteromer current could be partly overcome by specific disulfide cross-linking of oocyte-expressed P2X1 and P2X2 mutants resulting in non-functional P2X1/2Rs and their activation following reduction with dithiothreitol (Marquez-Klaka et al., 2009). However, the reduced agonist sensitivity in these mutants has to be considered. The fast desensitizing phenotype found for the P2X1/2 heteromer is somehow exceptional because in all other functionally described P2X heteromers the slowly desensitizing subunit determines the kinetic of the heteromer.

#### Evidence from native systems

The solid biochemical evidence for P2X1/2 heteromers is in contrast to the very limited evidence for this heteromer in native tissues. P2X1 and P2X2 subunits are widely distributed and overlap in many tissues (Burnstock and Knight, 2004). Transcripts of both subunits were found for example in vascular smooth muscle (Nori et al., 1998). However, generally, expression levels differed greatly, suggesting at most a small contribution of the respective heteromer. Whole-cell patch clamp and calcium imaging studies in mouse sympathetic neurons revealed a dominant P2X2-like phenotype and an αβ-meATP-sensitive receptor population that was largely absent in P2X1 knockout mice (Calvert and Evans, 2004). This αβ-meATP-sensitive current was blocked by Ca^2+^ and alkaline pH and it was concluded that it corresponds to a P2X1/2 heteromer. However, involvement of a third subunit was suggested since the current was not potentiated at acidic pH and showed a relatively slow time course of response.

### P2X1/4 heteromers

#### Biochemical evidence

Biochemical evidence for heteromerization between co-expressed P2X1 and P2X4 subunits was excluded in two co-immunoprecipitation studies performed in HEK cells (Le et al., 1998; Torres et al., 1999a), but was later found by co-purification and subsequent BN-PAGE analysis of *Xenopus* oocyte-expressed P2X1 and P2X4 subunits (Nicke et al., 2005).

#### Functional evidence

Functional evidence for the formation of P2X1/4 heteromers came from the generation of a new phenotype upon co-expression of P2X1 and P2X4 subunits in *Xenopus* oocytes (Nicke et al., 2005). The proposed P2X1/4 heteromer shares the moderately desensitizing kinetics and fast recovery upon repeated activation with the P2X4R. In contrast to the P2X4 homomer and in common with the P2X1 homomer, however, it is activated by low micromolar concentrations of αβ-meATP and inhibited by suramin and TNP-ATP. Although these currents were reproducibly found upon co-expression of both subunits, they were comparably small, suggesting that previous attempts to detect this heteromer failed due to preferential homomerization of both subunits.

#### Evidence from native systems

While heterologously expressed P2X4Rs are efficiently expressed as homotrimers in the plasma membrane, only few native receptors have been identified that resemble exactly the recombinant homomeric P2X4R, which is relatively insensitive to αβ-meATP (Coddou et al., 2011b). A possible explanation is that both P2X4R expression and its cycling between intracellular compartments and the plasma membrane are highly dynamic and a predominant localization of P2X4Rs in intracellular compartments has been described (e.g., Bobanovic et al., 2002; Qureshi et al., 2007; Stokes and Surprenant, 2009; Toulme et al., 2010). Thus, P2X4Rs appear to be expressed and/or translocated to the plasma membrane only under specific conditions (e.g., upon activation of microglial cells) and might require additional subunits to be stabilized at the plasma membrane. Receptors incorporating P2X4 subunits have been postulated in a wide variety of tissues but little evidence for a P2X1/4 heteromer in native tissues has been provided so far. The P2X4 subunit has the widest distribution pattern of all P2X subunits and overlap with P2X1 subunits is found in many tissues. For example, P2X1 and P2X4 subunits have been identified in different types of immune cells (Sim et al., 2007; Wareham et al., 2009; Woehrle et al., 2010b) and both subunits translocate to the immune synapse in stimulated T cells (Woehrle et al., 2010a). However, data from knockout mice (Sim et al., 2007) and pharmacological analysis (Wareham et al., 2009) indicate the presence of homomeric rather than heteromeric P2X1 and P2X4Rs in mouse macrophages and human mast cells, respectively. Co-expression of both subunits was also found by single cell RT-PCR in smooth muscle cells of renal resistance arteries (Harhun et al., 2010). Here, the characteristics of a current component that was insensitive to the P2X1 antagonist NF279 (Rettinger et al., 2000) were consistent with the properties of the P2X1/4 heteromer. A role of a P2X1/4 heteromer was also considered in neurogenic contractions in the guinea pig urinary bladder (Kennedy et al., 2007) and not excluded in coronary artery smooth muscle (Conant et al., 2008) and erythrocytes (Skals et al., 2009).

### P2X1/5 heteromers

#### Biochemical evidence

Biochemical evidence for the formation of heteromers consisting of P2X1 and P2X5 subunits was initially obtained from HEK293 cells transfected with epitope-tagged P2X1 and P2X5 subunits (Torres et al., 1998; Le et al., 1999). Here, the authors could demonstrate a direct association by co-purification. However, further biochemical evidence for P2X1 and P2X5 subunit interaction is still lacking in other *in vitro* models as well as in native tissues.

Several studies provide **functional evidence** for heteromerization of rat P2X1 and P2X5 subunits upon expression of both subunits in established expression systems like *Xenopus laevis* oocytes (Le et al., 1999; Rettinger et al., 2005) and cultured HEK293, CHO, and COS cells (Torres et al., 1998; Haines et al., 1999; Surprenant et al., 2000). The P2X1/5 heteromer shows distinct functional properties providing clear characteristics for its identification: (a) In contrast to the P2X1 homomer, its currents have a biphasic kinetic with a desensitizing peak and a non-desensitizing plateau current that are not desensitized by repeated agonist application. Depending on pH and ATP concentrations, the current response kinetics of the P2X1/5R differ (Surprenant et al., 2000). (b) It has a significant greater amplitude than the P2X5 homomer and, similar to the P2X1R, is activated by nanomolar ATP concentrations as well as by α,β-meATP (Torres et al., 1998; Haines et al., 1999; Le et al., 1999). (c) PPADS, suramin, and the specific P2X1R blocker NF449 antagonize with similar micromolar potencies the P2X1/5 heteromer and the P2X1 homomer (Haines et al., 1999; Rettinger et al., 2005), while the potency of TNP-ATP at the heteromer is markedly reduced in comparison to the P2X1 homomer and is more similar to that at the P2X5 homomer (Haines et al., 1999; Surprenant et al., 2000; Wildman et al., 2002). (d) A rebound current upon removal of agonist has been observed in some studies (Haines et al., 1999; Lalo et al., 2008). Peak currents of homomeric P2X5Rs are drastically decreased in a Ca^2+^ concentration dependent manner, whereas P2X1R responses are Ca^2+^ insensitive. Interestingly, increased extracellular Ca^2+^ significantly potentiated the steady-state currents of the P2X1/5 heteromer (Haines et al., 1999). Both acidification and alkalization have inhibitory effects on P2X1/5 heteromers while the homomeric receptors are only inhibited by an increased proton concentration (Surprenant et al., 2000).

#### Evidence from native systems

In contrast to the widely distributed expression of the P2X1 subunit, P2X5 subunit RNA has only been detected in a restricted number of tissues including the heart, sensory and motor neurons of the cervical spinal cord (Collo et al., 1996), vascular smooth muscle (Phillips and Hill, 1999), and astrocytes (Lalo et al., 2008). The presence of P2X1/5 heteromers was suggested in guinea-pig submucosal arterioles where an increase in spontaneous excitatory junction potentials and higher current amplitudes were measured following repetitive ATP application and increased Ca^2+^ concentration, respectively (Surprenant et al., 2000). The electrophysiological profile of this smooth muscle tissue supports rather the presence of P2X1/5 heteromers than P2X1 homomers that has been described in HEK293 cells expressing P2X1 and P2X5 subunits. In more recent studies, based on quantitative real time PCR and pharmacological characterization by whole cell voltage clamp experiments, evidence for P2X1/5Rs in acutely isolated cortical astrocytes was obtained (Lalo et al., 2008). Here, the heteromer appears to be involved in astrocytic excitability, which is driven by phosphoinositides and mediated through the lipid-binding domain of the P2X1 subunit (Ase et al., 2010).

### P2X2/3 heteromers

The P2X2/3 heteromer was the first one to be identified functionally and biochemically (Lewis et al., 1995; Radford et al., 1997). So far, it represents the best characterized P2X heteromer and solid evidence for its *in vivo* expression in sensory neurons and its functional role in sensory neurotransmission exists. As several valuable reviews regarding their presence in sensory and autonomic neurons and their physiological importance are available (North, 2002; Brederson and Jarvis, 2008; Burnstock, 2009a; Jarvis, 2010; Khakh and North, 2012), this chapter focuses only on some key findings and more recent studies.

#### Biochemical evidence and stoichiometry

Evidence for heteromerization of P2X2 and P2X3 subunits was first obtained from co-immunoprecipitation studies of P2X2 and P2X3 subunits heterologously expressed in Sf9 insect cells (Radford et al., 1997). Subsequent co-precipitation studies by Torres et al. (1999b) confirmed the interaction of P2X2 and P2X3 subunits in HEK cells and more recently, BN-PAGE analysis of oocyte-expressed affinity-tagged hP2X2 and hP2X3 subunits demonstrated that both subunits co-assemble in trimeric P2X2/3Rs, which are efficiently expressed at the plasma-membrane (Hausmann et al., 2012). By analysis of disulfide bond formations between engineered cysteine residues in P2X2 and P2X3 subunits a “head-to-tail” subunit arrangement was originally demonstrated (Jiang et al., 2003). Furthermore, this study revealed the presence of adjacent P2X3 subunits but not P2X2 subunits, indicating a P2X2(3)_2_ stoichiometry of P2X2/3 channels. This stoichiometry was further confirmed by co-expression and functional analysis of P2X2 or P2X3 subunits containing single or double mutated ATP binding sites (Wilkinson et al., 2006). The P2X2(3)_2_ stoichiometry is indirectly supported by the finding that the degree of potentiation by extracellular Zn^2+^ in the P2X2/3 heteromer was comparable to the limited Zn^2+^ effect seen with concatenated P2X2 trimers that contained only one wild type subunit with two Zn^2+^ binding site mutants (Nagaya et al., 2005).

#### Functional evidence

Functional evidence for the formation of P2X2/3 heteromers came initially from patch clamp analysis and comparison of rat nodose ganglia neurons and heterologously expressed P2X2 and P2X3 subunits (Lewis et al., 1995). This study revealed a non-desensitizing, αβ-meATP sensitive functional phenotype that could be unambiguously discriminated from the non-desensitizing α,β-meATP insensitive P2X2 and the fast desensitizing α,β-meATP sensitive P2X3 phenotypes. In addition, similar to the homomeric P2X2R, the heteromeric P2X2/3R is strongly potentiated at low pH values, while the homomeric P2X3R is much less pH sensitive and inhibited at low pH (King et al., 1996; Stoop et al., 1997; Wildman et al., 1999b). For a more detailed analysis of the pharmacologic and kinetic properties of heterologously expressed P2X2/3Rs see (Stoop et al., 1997; Virginio et al., 1998; Burgard et al., 2000; Liu et al., 2001; Spelta et al., 2002, 2003).

#### Evidence from native systems

Evidence from native systems led initially to the identification of heteromeric P2X2/3Rs; α,βme-ATP-elicited current responses in nodose ganglia neurons did not match those of any singly expressed P2X subunit but were reproduced by co-expression of P2X2 and P2X3 subunits (Lewis et al., 1995). Subsequently, convincing evidence for the existence of heteromeric P2X2/3 channels was also provided in sympathetic, trigeminal, and dorsal root ganglia neurons (Cook et al., 1997; Thomas et al., 1998; Burgard et al., 1999; Grubb and Evans, 1999; Ueno et al., 1999; Dunn et al., 2000, 2001; Lalo et al., 2001; Zhong et al., 2001; Petruska et al., 2002). Functional analysis of P2X2 and P2X2/P2X3 knockout mice further demonstrated/confirmed the presence of P2X2/3Rs in sensory and autonomic ganglia neurons and primary afferent nerve fibers in the urinary bladder (Cockayne et al., 2005) and defined the relative contribution of homomeric P2X2Rs and P2X3Rs and heteromeric P2X2/3Rs. This study revealed that ATP-induced currents in dorsal root ganglia (DRG) neurons are mediated by P2X3 and P2X2/3Rs, while those in nodose ganglion neurons are dominated by P2X2Rs and P2X2/3Rs. In sympathetic ganglion neurons, P2X3-containing receptors appear to be of minor functional importance and in the urinary bladder, P2X3Rs and P2X2/3Rs regulate urinary bladder reflexes. For more details about the role of these receptors in afferent sensory neurotransmission, mechanosensory transmission, and pain states see more recent reviews (Wirkner et al., 2007; Burnstock, 2009b; Jarvis, 2010; Burnstock et al., 2011; Khakh and North, 2012). Interestingly, a recent study by Serrano and colleagues showed that in contrast to rodent DRGs, P2X2 transcripts are virtually absent in human and monkey DRG neurons (Serrano et al., 2012). Hence, primate DRG neurons seem to be devoid of P2X2/3Rs and ATP-induced responses are mediated exclusively by P2X3Rs. This finding may significantly affect the translatability of rodent data to validate these receptors as targets for the treatment of pain. P2X2 and P2X3 subunits are also co-expressed in sensory nerve fibers in taste buds (Bo et al., 1999) and gustatory nerves and P2X2/P2X3 double-knockout mice exhibited abolished responses to taste stimuli (Finger et al., 2005; Eddy et al., 2009).

### P2X2/5 heteromers

#### Biochemical evidence

The rat P2X2/5 heteromer represents the most recent addition to the functionally characterized set of P2X subtypes. Apart from co-immunoprecipitation studies, biochemical evidence for its existence was provided by an ELISA assay that measured the increase in plasma membrane appearance of an HA-tagged trafficking-deficient P2X2 or P2X5 subunit by co-expression of the respective other subunit. In addition, their close spatial proximity was shown in bioluminescent resonance energy transfer studies and in bimolecular fluorescence complementation studies. In combination with cross-linking experiments using a membrane-impermeable cross-linker and native perfluorooctanoic acid (PFO)-PAGE analysis, these experiments indicated that P2X2/5 heterotrimers appear with both possible stoichiometries in the plasma membrane (Compan et al., 2012).

#### Functional evidence

Functional evidence for this heteromer was obtained upon co-expression of both subunits in *Xenopus* oocytes and HEK cells (Compan et al., 2012). Although the presence of homomeric P2X2Rs could not be prevented, these studies revealed a novel phenotype in oocytes with slightly reduced sensitivity to ATP, ATPγS, and BzATP. Interestingly, BzATP showed strongly reduced efficacy at this heteromer. Inhibition of the supposed heteromer by TNP-ATP was increased while αβ-meATP was ineffective at concentrations of 300 μM. Co-expression of both subunits in oocytes and/or HEK cells also produced ATP-activated time-dependent permeability changes for NMDG, YO-PRO-1, and ethidium that showed a clearly different time course compared to homomeric P2X2Rs and higher absolute fluorescence values or even no saturation. Most remarkably, HEK cells co-expressing both subunits displayed plasma membrane blebbing and flipping of phosphatidylserine from the inside surface of the plasma membrane to the outside surface (PS flip), two hallmark properties of the P2X7R that were not seen in cells expressing P2X2 subunits alone.

#### Evidence from native systems

Based on co-immunoprecipitation data from total brain and brain stem as well as immunohistochemistry data from dorsal root ganglia, spinal cord and trigeminal mesencephalic nucleus neurons in the mid pons, the presence of P2X2/5 heteromers in these tissues was suggested (Compan et al., 2012). However, the P2X2/5 heteromer differs from a previously characterized P2XR in trigeminal mesencephalic nucleus in its insensitivity to α,β-meATP (Patel et al., 2001).

### P2X2/6 heteromers

#### Biochemical evidence and stoichiometry

Evidence for the interaction of P2X2 and P2X6 subunits was initially obtained by co-immunoprecipitation (Torres et al., 1999a) and more recently, heterotrimerization of these subunits was confirmed by BN-PAGE analysis of oocyte expressed affinity-tagged hP2X2 and hP2X6 subunits (Hausmann et al., 2012). In contrast, to the heterotrimeric hP2X2/6 complex that is efficiently expressed at the plasma membrane, hP2X6 subunits do not form trimeric complexes (Aschrafi et al., 2004; Hausmann et al., 2012) and are retained in the ER. In HEK cells, co-expression of wt P2X6 subunits rescued ATP-elicited currents of P2X2 subunits harboring mutations in ATP-binding residues (such as K69A) that lead to the formation of non-functional homomeric P2X2Rs. Co-expression of wt P2X2 subunits with mutant P2X6 subunits and *vice versa* revealed that functional P2X2/6 heteromers consist of two P2X2 subunits and one P2X6 subunit. The P2X(2)_2_6 stoichiometry is in contrast to an AFM study of antibody-tagged isolated receptors, in which a variable subunit stoichiometry that depended on the relative subunit expression levels was observed (Barrera et al., 2007). However, in this study receptors purified from a crude membrane fraction, which also contained intracellular membranes, were analyzed.

#### Functional evidence

Functional evidence for the existence of a P2X2/6 heteromer was initially shown in *Xenopus* oocytes (King et al., 2000). Expression of P2X6 subunits alone did not produce functional channels. Co-expression of both subunits resulted in ATP-induced currents that had fast activating and slowly desensitizing kinetics similar to homomeric P2X2Rs, but showed a biphasic current decay upon removal of ATP. In a subset of cells, an additional transient current component was present that was never seen in oocytes expressing exclusively the P2X2 subunit. Another remarkable difference is the loss of the agonist activity of Ap_4_A, which is a full agonist at the P2X2R, but was almost inactive at the P2X2/6 heteromer (King et al., 2000). In contrast to homomeric P2X2Rs, which exhibit a current amplitude potentiation at high proton concentrations, heteromeric P2X2/6Rs showed a decreased current amplitude at acidic pH. In HEK cells, however, the co-expression of a four-fold excess of the P2X6 subunit with the P2X2 subunit revealed a current that was potentiated at decreasing pH values and that was not seen in HEK cells expressing exclusively P2X2 subunits (Hausmann et al., 2012). Thus, the cellular background might influence these properties.

#### Evidence from native systems

Co-expression of P2X2 and P2X6 transcripts has been described in some nuclei of the thalamus and hypothalamus and in specific laminae of the pineal gland (Collo et al., 1996). P2X2 and P2X6 subunits are also co-expressed in P19 embryonal carcinoma cells and neuronal stem cells (Resende et al., 2008; Schwindt et al., 2011). Ca^2+^-imaging-based pharmacological analysis revealed that P2X4Rs or P2X4-containing heteromultimers mediate ATP-induced calcium-responses of undifferentiated P19 embryonal carcinoma cells, while P2X2Rs and possibly heteromeric P2X2/6Rs are the major mediators of calcium-responses in neuronally differentiated cells after retinoic acid treatment (Resende et al., 2008). However, it remained partially unresolved whether the ATP-induced calcium transients of neuronally differentiated P19 cells are mediated by homomeric P2X2 or heteromeric P2X2/6Rs (Resende et al., 2008), although an simultaneous upregulation of the P2X2 and P2X6 expression was shown during neuronal differentiation (Resende et al., 2007). Also, during neuronal differentiation of neuronal progenitor cells of neurospheres the expression of P2X2 and P2X6 subunits was upregulated (Schwindt et al., 2011). In summary, although P2X2 and P2X6 are co-expressed in several tissues and their simultaneous regulation during neuronal differentiation was shown, there is to our knowledge no clear and direct evidence for the presence of functional heterotrimeric P2X2/6 assemblies in native cells or tissues.

### P2X4/6 heteromers

#### Biochemical evidence

Biochemical evidence for the existence of P2X4/6 heteromers was initially demonstrated by co-purification of epitope-tagged P2X4 and P2X6 subunits upon expression in HEK293 cells (Le et al., 1998; Torres et al., 1999a). There is a controversy regarding the ability of the P2X6 subtype to form homotrimers, since its expression in *in vitro* models failed to generate a functional receptor (Soto et al., 1996b; Le et al., 1998; Khakh et al., 1999; Torres et al., 1999a; King et al., 2000; Aschrafi et al., 2004). It has been suggested that the P2X6 subtype needs to get posttranslationally glycosylated to form a functional homomeric receptor in HEK293 cells (Jones et al., 2004). However, endogenous P2X4Rs have been reported in HEK293 cells (Worthington et al., 1999) (own unpublished observations) and could promote P2X6 subunit assembly and trafficking in form of heteromers. Using AFM in combination with surface biotinylation and immunofluorescence analysis, it has been shown that P2X6 subunits expressed in NRK cells do not form homotrimers. Substitution of 14 uncharged N-terminal amino acid residues by charged residues increased glycosylation and plasma membrane insertion of P2X6 subunits, indicating that their ER release is inhibited by this uncharged N-terminal region (Ormond et al., 2006). Co-immunoprecipitation and immunofluorescent studies in HEK293 cells showed a preferred assembly of P2X4 subunits into heterotrimeric P2X4/6Rs (Le et al., 1998; Bobanovic et al., 2002) and regulation of the P2X4/6 heteromer trafficking by the P2X4 subunit. The homomeric P2X4R showed an intracellular punctate distribution with sparse localization at the plasma membrane. In contrast, the GFP-tagged P2X6 subunit was diffusely distributed intracellularly and co-localized with the ER marker calreticulin, without any indication for the presence of the P2X6R at the cell surface. However, in the presence of the P2X4 subunit, the P2X6 subunit showed a similar punctate pattern as the homomeric P2X4R (Bobanovic et al., 2002).

#### Functional evidence

The functional differentiation between homomeric P2X4 and heteromeric P2X4/6 assemblies is difficult, since the biophysical and pharmacological differences are not that clear and controverse data exist regarding the functional expression and αβ-meATP sensitivity of the P2X6R (Collo et al., 1996; Jones et al., 2004). The sensitivity of homomeric P2X4Rs to the partial agonist αβ-meATP is relatively low and species-dependent with EC_50_ values of 7 and 19.2 μM for mouse and human receptors, respectively, whereas rat P2X4Rs showed only very weak responses (Jones et al., 2000). Upon co-expression of P2X4 and P2X6 subunits in *Xenopus* oocytes, receptors were observed that showed (1) a three to five fold higher sensitivity to the partial agonists αβ-meATP and 2MeSATP than the P2X4R, (2) an enhanced inhibition by PPADS, suramin, and RB-2, and (3) peak current amplitudes that differed from those obtained by expression of the P2X4 subunit alone. However, current kinetics and current potentiation by IVM, as well as the effects of modulators like protons and zinc ions were undistinguishable between P2X4 homomers and P2X4/6 heteromers (Le et al., 1998; Khakh et al., 1999).

#### Evidence from native systems

P2X4 and P2X6 RNAs are abundant and show a broad overlapping distribution throughout the central nervous system with significant amounts in the hippocampus and the cerebellum (Collo et al., 1996; Soto et al., 1996a,b). However, the function of homomeric P2X4 or heteromeric P2X4/6Rs in neurons remains unclear. Immunogold-labeling demonstrated the presence of P2X4 and P2X6 subunits in perikarya and dendritic spines of cerebellar Purkinje and hippocampal, pyramidal CA1 neurons, where they were suggested to form heteromeric assemblies (Rubio and Soto, 2001). Although P2X4 transcripts were found in hippocampal CA1 pyramidal neurons, αβ-meATP induced responses from most CA1 neurons remained unaltered upon IVM treatment, indicating that neither P2X4 homomers nor P2X4/6 heteromers are expressed in these cells (Khakh et al., 1999). However, a possible role of the P2X4 subunit in regulating synaptic plasticity was suggested based on studies with P2X4 knockout mice. The application of IVM on wt-mouse brain slices potentiated the EPSPs from CA1 neurons, whereas IVM had no effect in P2X4 knockout mice, which also showed an impaired long-term potentiation in the hippocampus (Sim et al., 2006). Early studies on rat hippocampal CA3 neurons revealed αβ-meATP-activated responses that were blocked by suramin, but were insensitive to PPADS (Ross et al., 1998), ruling out a contribution of P2X4Rs or P2X4/6Rs to neuronal excitation in these neurons. These findings were confirmed by later studies reporting that IVM had no effect on these cells (Mori et al., 2001; Kondratskaya et al., 2008). However, a significant contribution of P2X4 subunit-containing receptors to the generation of EPSPs was shown in neocortical neurons, where ATP- and αβ-meATP-mediated current responses were potentiated by IVM, but were not inhibited by PPADS (Lalo et al., 2007).

According to functional and biochemical analyses, P2X4 and P2X6 subunits are endogenously expressed in P19 murine embryonal carcinoma cells and show varying expression levels during the non-differentiated and neuronal progenitor states, suggesting a possible role for heteromeric P2X4/6Rs in regulation and induction of neurogenesis (Resende et al., 2007, 2008). P2X4 and P2X6 subtype expression was also found in human non-cystic and cystic fibrosis epithelial cells. The knockdown of the P2X6 subunit by siRNA in these cells resulted in a significant attenuation of Zn^2+^-mediated Ca^2+^ influx, which was similar to the effect that occurred upon P2X4 subunit knockdown (Liang et al., 2005). Therefore, it was suggested that P2X4/6Rs play a role in the regulation of the Ca^2+^ influx that restores Cl^−^ secretion across the airway epithelia. Immunofluorescent stainings on human umbilicial endothelial cells revealed a colocalization of P2X4 and P2X6 subunits with VE-cadherin in cellular junctions, where they were suggested to be involved in the modulation of cell-cell adhesion processes via the mediation of Ca^2+^ signaling (Glass et al., 2002).

### P2X4/7 heteromers

P2X4R and P2X7R genes are located next to each other on human and rat chromosome 12 and murine chromosome 5. Both subunits share a high sequence similarity and show overlapping distribution in many tissues such as epithelia, endothelia, and immune cells.

#### Biochemical evidence

Biochemical evidence for an association between P2X4 and P2X7 subunits was originally provided by co-immunoprecipitation from transfected HEK cells and from bone marrow-derived macrophages (Guo et al., 2007). Several subsequent studies, however, failed to confirm an association between both subunits within heterotrimeric complexes: BN-PAGE analysis of P2X7 complexes from various native tissues (bone marrow, lymph node, salivary gland) that contained both subunits revealed exclusively complexes that corresponded in size to the homomeric P2X7 complex, which can be differentiated by its size from any heteromeric P2X7-containing complex (Nicke, 2008). Likewise, BN-PAGE analysis and cross-linking data from primary cultures of rat macrophages and mouse microglia revealed an interaction between homomeric P2X7 and P2X4 complexes rather than P2X4/7 heteromers (Boumechache et al., 2009). This study further showed that P2X4Rs were predominantly intracellular, whereas P2X7Rs were mainly localized to the plasma membrane. However, both complexes could be co-immunoprecipitated. Co-expression of P2X4 and P2X7 subunits in NRK cells increased the surface expression of P2X4 subunits about two-fold (Guo et al., 2007). A pairing of P2X4 and P2X7 (and also P2X2 and P2X4) homotrimers was supported by cross-linking analysis and AFM imaging of HEK cell-expressed receptors (Antonio et al., 2011). In conclusion, there is currently no biochemical evidence for the presence of heterotrimeric P2X7Rs. It has to be noted, however, that P2X2 and P2X4 receptors have been found to be rather “sticky” proteins that appear to associate into higher aggregates more easily than other subunits (Aschrafi et al., 2004; Weinhold et al., 2010) (observation from the authors labs) and also P2X1 trimers appear to associate into dimers and higher complexes under certain conditions (Nicke, 2008). Whether this represents an expression-level and/or detergent-dependent artefact or is the result of a specific interaction is not clear at present. In the mouse alveolar epithelial E10 cell line, P2X4 and P2X7 receptors were shown to associate partly with lipid rafts and the P2X7R was found to interact with caveolin 1 (Barth et al., 2008). Co-immunoprecipitation, high resolution clear native PAGE, and BN-PAGE data suggest that all three proteins can be constituents of higher order protein complexes in these cells, but that caveolin 1 interacts only with P2X7Rs directly. In support of this, knock down by shRNAs demonstrated that downregulation of P2X7 subunits affects protein levels, localization, and complex organization of both caveolin 1 and P2X4. In contrast, P2X4 knock-down affected only P2X7 protein levels and localization but not caveolin 1 (Weinhold et al., 2010). In both cases, upregulation of the respective other P2X subunit was found. In the kidney, however, a mutual negative influence on subunit expression levels was found (Craigie et al., 2013). Using the respective knockout mice, it was shown that ablation of one subunit significantly reduced the mRNA levels of the respective other subunit.

#### Functional evidence for mutual interactions

Functional evidence for mutual interactions between P2X4 and P2X7 subunits and/or receptors has been found in HEK cells co-transfected with both subunits (Guo et al., 2007; Casas-Pruneda et al., 2009). Thus, co-expression of a dominant negative P2X4 subunit was found to reduce the P2X7R currents without reducing their number in the plasma membrane and to confer IVM and TNP-ATP sensitivity as well as some BBG resistance to the P2X7R (Guo et al., 2007). In another study, the ATP-activated current decay in TEA^+^-containing solution was accelerated by co-expression of P2X4 subunits, which themselves showed no current under these ionic conditions. Furthermore, ethidium uptake was slowed down and decreased, a P2X fraction with lower ATP-sensitivity was observed, and a concentration-dependent lack of potentiation by IVM was seen in the presence of P2X4 subunits (Casas-Pruneda et al., 2009).

#### Evidence from native systems

Co-expression of mouse P2X4 and P2X7 subunits in HEK cells reproduced the properties of ATP-activated currents in parotid acinar cells better than expression of each subunit alone (Casas-Pruneda et al., 2009). In freshly isolated rabbit airway ciliated cells an ATP-gated cation channel has been characterized (Ma et al., 2006) that shares the following properties with the P2X7R but not the P2X4R: (a) low sensitivity to ATP, (b) modulation by external Na^+^, (c) inhibition by extracellular divalent cations as well as (d) sensitivity to the P2X7 antagonists BBG and KN-62. However, in contrast to the P2X7R, this P2X(cilia) did not show pore dilation and, in agreement with P2X4R-associated properties, its current was augmented by Zn^2+^ and IVM. A mixture of both homomeric channels was excluded because the dose response curve of this channel could be described by a simple Hill equation and a P2X4/7R heteromer or a modified P2X4R were proposed as possible explanations. In support of a homomeric P2X7R, however, it was recently found that IVM also species-specifically potentiates P2X7R current amplitudes in a very similar manner as described for the P2X(cilia) (Casas-Pruneda et al., 2009; Nörenberg et al., 2010), see also Surprenant and North (2009). Adding to the complexity of P2X7R characterization, polymorphisms have been identified in human and mouse P2X7 isoforms that influence their ability to form dye permeable pores, at least when studied in native cell preparations (Gu et al., 2001; Adriouch et al., 2002; Le Stunff et al., 2004; Sorge et al., 2012). It has to be noted, however, that no obvious functional differences were found if the respective recombinant channels were studied (Boldt et al., 2003; Donnelly-Roberts et al., 2009; Schwarz et al., 2012; Xu et al., 2012), suggesting the involvement of cell-specific factors in pore formation and/or assay-dependent/methodological differences in these studies. Thus, species-specific differences in pharmacology as well as polymorphisms need to be considered when characterizing P2X7Rs in native tissues.

Functional interactions between both receptors were recently also shown in mouse macrophage RAW246.7 cells, where P2X4 knock down by shRNA suppressed ATP-induced cell death and release of HMGB1 and Il1β, and facilitated the production of reactive oxygen species. However, P2X4 subunit knock down did not affect P2X7-mediated pore formation and MAPK signaling (Kawano et al., 2012a,b). From similar experiments, it was suggested that the P2X4R positively modulates P2X7-dependent cytokine release from bone marrow-derived dendritic cells (Sakaki et al., 2013). Most recently, a supposedly P2X4-containing receptor was described in murine myenteric neurons. Compared to the homomeric P2X4R this receptor had lower ATP sensitivity but increased sensitivity to the antagonists PPADS and suramin and its current was potentiated by IVM. Since P2X2 and P2X7 subunits had also been reported to be expressed in these cells, the possibility of P2X2/4/7 heteromers was considered (Maria et al., 2013).

### Evidence for clustering of trimeric P2XRs

For heterologously expressed P2X2Rs it was observed that properties such as mean open times, open channel noise, potentiation by Zn^2+^ and pH, the EC_50_ value for ATP, the ability to form large pores, and the inward rectification depend on the P2X2 expression level or density (Ding and Sachs, 2002; Clyne et al., 2003; Fujiwara and Kubo, 2004), suggesting interactions between homotrimeric P2X2Rs. A physical interaction between P2X2Rs is supported by biochemical evidence for an increased tendency of this receptor to form higher order complexes (Aschrafi et al., 2004) and high densities of GFP-tagged P2X2Rs could be observed upon their activation-dependent clustering in embryonic hippocampal neurons (Khakh et al., 2001). In addition, physical interactions between homotrimeric P2X7 and P2X4 receptors, and P2X2 and P2X4 receptors have been observed (Boumechache et al., 2009; Antonio et al., 2011). Whether these interactions are direct or via clustering molecules or within lipid domains and whether they have physiological relevance or represent overexpression artefacts remains to be determined. In this context, it is interesting that P2X4 and P2X7 were co-precipitated with the extracellular matrix component biglycan and soluble biglycan-induced clustering of P2X4 and P2X7 receptors with Toll-like receptor (TLR) 2/4 was found to underlie the activation of the inflammasome by this component (Babelova et al., 2009). Functional and physical interactions between P2XRs and other ion channels (e.g., members of the Cys-loop receptor and epithelial Na^+^ channel families) have also been described [for a recent review see (Kaczmarek-Hajek et al., 2012)].

## Summary and outlook

P2XRs contain three intersubunit binding sites for orthosteric ligands. In heteromeric receptors all three binding sites differ significantly and thus offer theoretically the possibility for subtype-specific targeting of heteromers. However, proof of this concept is still lacking.

For heterologously expressed receptors, a combination of pharmacology and current kinetics in most cases allowed the discrimination of heteromeric receptor responses from those of homomeric receptors. However, identification of matching responses in native cells proved difficult and was only convincingly achieved in case of the P2X1/5 and P2X2/3 heteromers. Thus, it is likely that additional factors such as receptor modifications (e.g., phosphorylation), P2X splice variants, interacting proteins, P2XR clustering, the physiological expression background, expression levels, and trafficking influence P2XR properties under native conditions.

Co-immunoprecipitation still represents one of the standard experiments to proof a physical interaction between proteins. However, several pitfalls need to be considered: First, the method does not allow to differentiate between association of trimeric receptors and heterotrimerization of subunits. Second, it is difficult to exclude that the observed interaction is due to artificial aggregation of the overexpressed protein or occurs artificially during the solubilization and purification process, which is generally optimized toward the detection of an interaction. Third, if performed in native tissues, this method also critically depends on the availability of reliable antibodies. Thus, methods for the direct visualization of complexes and biochemical and/or functional assays, which are able to detect interactions within the membrane, are needed and experiments should preferably be performed on native preparations or at physiological expression levels. With RNA knockdown technology, knockout animals, and transgenic animals expressing tagged P2XRs, suitable control experiments can be performed and in combination with the increasingly available subtype selective compounds, knockout animals provide valuable models to decipher the composition of heteromeric complexes by pharmacological means.

### Conflict of interest statement

The authors declare that the research was conducted in the absence of any commercial or financial relationships that could be construed as a potential conflict of interest.

## References

[B1] Acuna-Castillo C. Coddou C. Bull P. Brito J. Huidobro-Toro J. P. (2007). Differential role of extracellular histidines in copper, zinc, magnesium and proton modulation of the P2X7 purinergic receptor. J. Neurochem. 101, 17–26 10.1111/j.1471-4159.2006.04343.x 17394459

[B2] Adriouch S. Dox C. Welge V. Seman M. Koch-Nolte F. Haag F. (2002). Cutting edge: a natural P451L mutation in the cytoplasmic domain impairs the function of the mouse P2X7 receptor. J. Immunol. 169, 4108–4112 12370338 10.4049/jimmunol.169.8.4108

[B3] Agresti C. Meomartini M. E. Amadio S. Ambrosini E. Serafini B. Franchini L. (2005). Metabotropic P2 receptor activation regulates oligodendrocyte progenitor migration and development. Glia 50, 132–144 10.1002/glia.20160 15657938

[B4] Allsopp R. C. El Ajouz S. Schmid R. Evans R. J. (2011). Cysteine scanning mutagenesis (residues Glu52-Gly96) of the human P2X1 receptor for ATP: mapping agonist binding and channel gating. J. Biol. Chem. 286, 29207–29217 10.1074/jbc.M111.260364 21690089 PMC3190727

[B5] Anderson C. M. Nedergaard M. (2006). Emerging challenges of assigning P2X7 receptor function and immunoreactivity in neurons. Trends Neurosci. 29, 257–262 10.1016/j.tins.2006.03.003 16564580

[B6] Antonio L. S. Stewart A. P. Xu X. J. Varanda W. A. Murrell-Lagnado R. D. Edwardson J. M. (2011). P2X4 receptors interact with both P2X2 and P2X7 receptors in the form of homotrimers. Br. J. Pharmacol. 163, 1069–1077 10.1111/j.1476-5381.2011.01303.x 21385174 PMC3130952

[B7] Aschrafi A. Sadtler S. Niculescu C. Rettinger J. Schmalzing G. (2004). Trimeric architecture of homomeric P2X2 and heteromeric P2X1+2 receptor subtypes. J. Mol. Biol. 342, 333–343 10.1016/j.jmb.2004.06.092 15313628

[B8] Ase A. R. Bernier L. P. Blais D. Pankratov Y. Seguela P. (2010). Modulation of heteromeric P2X1/5 receptors by phosphoinositides in astrocytes depends on the P2X1 subunit. J. Neurochem. 113, 1676–1684 10.1111/j.1471-4159.2010.06734.x 20374427

[B9] Babelova A. Moreth K. Tsalastra-Greul W. Zeng-Brouwers J. Eickelberg O. Young M. F. (2009). Biglycan, a danger signal that activates the NLRP3 inflammasome via toll-like and P2X receptors. J. Biol. Chem. 284, 24035–24048 10.1074/jbc.M109.014266 19605353 PMC2781998

[B10] Baqi Y. Hausmann R. Rosefort C. Rettinger J. Schmalzing G. Muller C. E. (2011). Discovery of potent competitive antagonists and positive modulators of the P2X2 receptor. J. Med. Chem. 54, 817–830 10.1021/jm1012193 21207957

[B11] Barrera N. P. Henderson R. M. Murrell-Lagnado R. D. Edwardson J. M. (2007). The stoichiometry of P2X2/6 receptor heteromers depends on relative subunit expression levels. Biophys. J. 93, 505–512 10.1529/biophysj.106.101048 17449665 PMC1896263

[B12] Barrera N. P. Ormond S. J. Henderson R. M. Murrell-Lagnado R. D. Edwardson J. M. (2005). Atomic force microscopy imaging demonstrates that P2X2 receptors are trimers but that P2X6 receptor subunits do not oligomerize. J. Biol. Chem. 280, 10759–10765 10.1074/jbc.M412265200 15657042

[B13] Barth K. Weinhold K. Guenther A. Linge A. Gereke M. Kasper M. (2008). Characterization of the molecular interaction between caveolin-1 and the P2X receptors 4 and 7 in E10 mouse lung alveolar epithelial cells. Int. J. Biochem. Cell Biol. 40, 2230–2239 10.1016/j.biocel.2008.03.001 18407780

[B14] Bean B. P. (1990). ATP-activated channels in rat and bullfrog sensory neurons: concentration dependence and kinetics. J. Neurosci. 10, 1–10 1688928 10.1523/JNEUROSCI.10-01-00001.1990 PMC6570325

[B15] Bean B. P. (1992). Pharmacology and electrophysiology of ATP-activated ion channels. Trends Pharmacol. Sci. 13, 87–90 10.1016/0165-6147(92)90032-2 1374198

[B16] Bhargava Y. Rettinger J. Mourot A. (2012). Allosteric nature of P2X receptor activation probed by photoaffinity labelling. Br. J. Pharmacol. 167, 1301–1310 10.1111/j.1476-5381.2012.02083.x 22725669 PMC3504995

[B17] Bo X. Alavi A. Xiang Z. Oglesby I. Ford A. Burnstock G. (1999). Localization of ATP-gated P2X2 and P2X3 receptor immunoreactive nerves in rat taste buds. Neuroreport 10, 1107–1111 10.1097/00001756-199904060-00037 10321492

[B18] Bo X. Jiang L. H. Wilson H. L. Kim M. Burnstock G. Surprenant A. (2003). Pharmacological and biophysical properties of the human P2X5 receptor. Mol. Pharmacol. 63, 1407–1416 10.1124/mol.63.6.1407 12761352

[B19] Bobanovic L. K. Royle S. J. Murrell-Lagnado R. D. (2002). P2X receptor trafficking in neurons is subunit specific. J. Neurosci. 22, 4814–4824 12077178 10.1523/JNEUROSCI.22-12-04814.2002 PMC6757758

[B20] Bodnar M. Wang H. Riedel T. Hintze S. Kato E. Fallah G. (2011). Amino acid residues constituting the agonist binding site of the human P2X3 receptor. J. Biol. Chem. 286, 2739–2749 10.1074/jbc.M110.167437 21098022 PMC3024770

[B21] Boldt W. Klapperstuck M. Buttner C. Sadtler S. Schmalzing G. Markwardt F. (2003). Glu496Ala polymorphism of human P2X7 receptor does not affect its electrophysiological phenotype. Am. J. Physiol. Cell Physiol. 284, C749–C756 10.1152/ajpcell.00042.2002 12431909

[B22] Boumechache M. Masin M. Edwardson J. M. Gorecki D. C. Murrell-Lagnado R. (2009). Analysis of assembly and trafficking of native P2X4 and P2X7 receptor complexes in rodent immune cells. J. Biol. Chem. 284, 13446–13454 10.1074/jbc.M901255200 19304656 PMC2679444

[B23] Brake A. J. Wagenbach M. J. Julius D. (1994). New structural motif for ligand-gated ion channels defined by an ionotropic ATP receptor. Nature 371, 519–523 10.1038/371519a0 7523952

[B24] Brederson J. D. Jarvis M. F. (2008). Homomeric and heteromeric P2X3 receptors in peripheral sensory neurons. Curr. Opin. Investig. Drugs 9, 716–725 18600577

[B25] Brotherton-Pleiss C. E. Dillon M. P. Ford A. P. Gever J. R. Carter D. S. Gleason S. K. (2010). Discovery and optimization of RO-85, a novel drug-like, potent, and selective P2X3 receptor antagonist. Bioorg. Med. Chem. Lett. 20, 1031–1036 10.1016/j.bmcl.2009.12.044 20045645

[B26] Brown S. G. Townsend-Nicholson A. Jacobson K. A. Burnstock G. King B. F. (2002). Heteromultimeric P2X(1/2) receptors show a novel sensitivity to extracellular pH. J. Pharmacol. Exp. Ther. 300, 673–680 10.1124/jpet.300.2.673 11805232 PMC5577565

[B27] Browne L. E. Jiang L. H. North R. A. (2010). New structure enlivens interest in P2X receptors. Trends Pharmacol. Sci. 31, 229–237 10.1016/j.tips.2010.02.004 20227116 PMC2954318

[B28] Burgard E. C. Niforatos W. Van Biesen T. Lynch K. J. Kage K. L. Touma E. (2000). Competitive antagonism of recombinant P2X(2/3) receptors by 2′, 3′-O-(2,4,6-trinitrophenyl) adenosine 5′-triphosphate (TNP-ATP). Mol. Pharmacol. 58, 1502–1510 10.1124/mol.58.6.1502 11093790

[B29] Burgard E. C. Niforatos W. Van Biesen T. Lynch K. J. Touma E. Metzger R. E. (1999). P2X receptor-mediated ionic currents in dorsal root ganglion neurons. J Neurophysiol 82, 1590–1598 10482772 10.1152/jn.1999.82.3.1590

[B30] Burnstock G. (2009a). Purinergic mechanosensory transduction and visceral pain. Mol. Pain 5, 69 10.1186/1744-8069-5-69 19948030 PMC2789721

[B31] Burnstock G. (2009b). Purinergic receptors and pain. Curr. Pharm. Des. 15, 1717–1735 10.2174/138161209788186335 19442186

[B32] Burnstock G. Knight G. E. (2004). Cellular distribution and functions of P2 receptor subtypes in different systems. Int. Rev. Cytol. 240, 31–304 10.1016/S0074-7696(04)40002-3 15548415

[B33] Burnstock G. Krugel U. Abbracchio M. P. Illes P. (2011). Purinergic signalling: from normal behaviour to pathological brain function. Prog. Neurobiol. 95, 229–274 10.1016/j.pneurobio.2011.08.006 21907261

[B34] Calvert J. A. Evans R. J. (2004). Heterogeneity of P2X receptors in sympathetic neurons: contribution of neuronal P2X1 receptors revealed using knockout mice. Mol. Pharmacol. 65, 139–148 10.1124/mol.65.1.139 14722245

[B35] Casas-Pruneda G. Reyes J. P. Perez-Flores G. Perez-Cornejo P. Arreola J. (2009). Functional interactions between P2X4 and P2X7 receptors from mouse salivary epithelia. J. Physiol. 587, 2887–2901 10.1113/jphysiol.2008.167395 19403602 PMC2718248

[B36] Clyne J. D. Brown T. C. Hume R. I. (2003). Expression level dependent changes in the properties of P2X2 receptors. Neuropharmacology 44, 403–412 10.1016/S0028-3908(02)00406-9 12604087

[B37] Clyne J. D. Lapointe L. D. Hume R. I. (2002). The role of histidine residues in modulation of the rat P2X(2) purinoceptor by zinc and pH. J. Physiol. 539, 347–359 10.1113/jphysiol.2001.013244 11882669 PMC2290168

[B38] Cockayne D. A. Dunn P. M. Zhong Y. Rong W. Hamilton S. G. Knight G. E. (2005). P2X2 knockout mice and P2X2/P2X3 double knockout mice reveal a role for the P2X2 receptor subunit in mediating multiple sensory effects of ATP. J. Physiol. 567, 621–639 10.1113/jphysiol.2005.088435 15961431 PMC1474198

[B39] Cockayne D. A. Hamilton S. G. Zhu Q. M. Dunn P. M. Zhong Y. Novakovic S. Ford A. P. (2000). Urinary bladder hyporeflexia and reduced pain-related behaviour in P2X3-deficient mice. Nature 407, 1011–1015 10.1038/35039519 11069181

[B40] Coddou C. Acuna-Castillo C. Bull P. Huidobro-Toro J. P. (2007). Dissecting the facilitator and inhibitor allosteric metal sites of the P2X4 receptor channel: critical roles of CYS132 for zinc potentiation and ASP138 for copper inhibition. J. Biol. Chem. 282, 36879–36886 10.1074/jbc.M706925200 17962187

[B41] Coddou C. Morales B. Gonzalez J. Grauso M. Gordillo F. Bull P. (2003). Histidine 140 plays a key role in the inhibitory modulation of the P2X4 nucleotide receptor by copper but not zinc. J. Biol. Chem. 278, 36777–36785 10.1074/jbc.M305177200 12819199

[B42] Coddou C. Stojilkovic S. S. Huidobro-Toro J. P. (2011a). Allosteric modulation of ATP-gated P2X receptor channels. Rev. Neurosci. 22, 335–354 10.1515/rns.2011.014 21639805 PMC3647606

[B43] Coddou C. Yan Z. Obsil T. Huidobro-Toro J. P. Stojilkovic S. S. (2011b). Activation and regulation of purinergic P2X receptor channels. Pharmacol. Rev. 63, 641–683 10.1124/pr.110.003129 21737531 PMC3141880

[B44] Collo G. North R. A. Kawashima E. Merlo-Pich E. Neidhart S. Surprenant A. (1996). Cloning OF P2X5 and P2X6 receptors and the distribution and properties of an extended family of ATP-gated ion channels. J. Neurosci. 16, 2495–2507 8786426 10.1523/JNEUROSCI.16-08-02495.1996 PMC6578782

[B45] Colquhoun D. (1998). Binding, gating, affinity and efficacy: the interpretation of structure-activity relationships for agonists and of the effects of mutating receptors. Br. J. Pharmacol. 125, 924–947 10.1038/sj.bjp.0702164 9846630 PMC1565672

[B46] Compan V. Ulmann L. Stelmashenko O. Chemin J. Chaumont S. Rassendren F. (2012). P2X2 and P2X5 subunits define a new heteromeric receptor with P2X7-like properties. J. Neurosci. 32, 4284–4296 10.1523/JNEUROSCI.6332-11.2012 22442090 PMC6621234

[B47] Conant A. R. Theologou T. Dihmis W. C. Simpson A. W. (2008). Diadenosine polyphosphates are selective vasoconstrictors in human coronary artery bypass grafts. Vascul. Pharmacol. 48, 157–164 10.1016/j.vph.2008.01.005 18325842

[B48] Cook S. P. Vulchanova L. Hargreaves K. M. Elde R. McCleskey E. W. (1997). Distinct ATP receptors on pain-sensing and stretch-sensing neurons. Nature 387, 505–508 10.1038/387505a0 9168113

[B49] Coutinho-Silva R. Ojcius D. M. Gorecki D. C. Persechini P. M. Bisaggio R. C. Mendes A. N. (2005). Multiple P2X and P2Y receptor subtypes in mouse J774, spleen and peritoneal macrophages. Biochem. Pharmacol. 69, 641–655 10.1016/j.bcp.2004.11.012 15670583

[B50] Cox J. A. Barmina O. Voigt M. M. (2001). Gene structure, chromosomal localization, cDNA cloning and expression of the mouse ATP-gated ionotropic receptor P2X5 subunit. Gene 270, 145–152 10.1016/S0378-1119(01)00484-X 11404011

[B51] Craigie E. Birch R. E. Unwin R. J. Wildman S. S. (2013). The relationship between P2X4 and P2X7: a physiologically important interaction? Front. Physiol. 4:216 10.3389/fphys.2013.00216 23966951 PMC3744038

[B52] Da Silva R. L. Resende R. R. Ulrich H. (2007). Alternative splicing of P2X6 receptors in developing mouse brain and during *in vitro* neuronal differentiation. Exp. Physiol. 92, 139–145 10.1113/expphysiol.2006.921304 17259301

[B53] Diaz-Hernandez M. Cox J. A. Migita K. Haines W. Egan T. M. Voigt M. M. (2002). Cloning and characterization of two novel zebrafish P2X receptor subunits. Biochem. Biophys. Res. Commun. 295, 849–853 10.1016/S0006-291X(02)00760-X 12127972

[B54] Ding S. Sachs F. (1999). Single channel properties of P2X2 purinoceptors. J. Gen. Physiol. 113, 695–720 10.1085/jgp.113.5.695 10228183 PMC2222910

[B55] Ding S. Sachs F. (2000). Inactivation of P2X2 purinoceptors by divalent cations. J. Physiol. 522(Pt 2), 199–214 10.1111/j.1469-7793.2000.t01-1-00199.x 10639098 PMC2269756

[B56] Ding S. Sachs F. (2002). Evidence for non-independent gating of P2X2 receptors expressed in Xenopus oocytes. BMC Neurosci. 3:17 10.1186/1471-2202-3-17 12421468 PMC137587

[B57] Donnelly-Roberts D. L. Namovic M. T. Han P. Jarvis M. F. (2009). Mammalian P2X7 receptor pharmacology: comparison of recombinant mouse, rat and human P2X7 receptors. Br. J. Pharmacol. 157, 1203–1214 10.1111/j.1476-5381.2009.00233.x 19558545 PMC2743839

[B58] Donnelly-Roberts D. McGaraughty S. Shieh C. C. Honore P. Jarvis M. F. (2008). Painful purinergic receptors. J. Pharmacol. Exp. Ther. 324, 409–415 10.1124/jpet.106.105890 18042830

[B59] Du J. Dong H. Zhou H. X. (2012a). Gating mechanism of a P2X4 receptor developed from normal mode analysis and molecular dynamics simulations. Proc. Natl. Acad. Sci. U.S.A. 109, 4140–4145 10.1073/pnas.1119546109 22378652 PMC3306669

[B60] Du J. Dong H. Zhou H. X. (2012b). Size matters in activation/inhibition of ligand-gated ion channels. Trends Pharmacol. Sci. 33, 482–493 10.1016/j.tips.2012.06.005 22789930 PMC3427461

[B61] Duckwitz W. Hausmann R. Aschrafi A. Schmalzing G. (2006). P2X5 subunit assembly requires scaffolding by the second transmembrane domain and a conserved aspartate. J. Biol. Chem. 281, 39561–39572 10.1074/jbc.M606113200 17001079

[B62] Dunn P. M. Liu M. Zhong Y. King B. F. Burnstock G. (2000). Diinosine pentaphosphate: an antagonist which discriminates between recombinant P2X(3) and P2X(2/3) receptors and between two P2X receptors in rat sensory neurones. Br. J. Pharmacol. 130, 1378–1384 10.1038/sj.bjp.0703404 10903979 PMC1572177

[B63] Dunn P. M. Zhong Y. Burnstock G. (2001). P2X receptors in peripheral neurons. Prog. Neurobiol. 65, 107–134 10.1016/S0301-0082(01)00005-3 11403876

[B64] Eddy M. C. Eschle B. K. Barrows J. Hallock R. M. Finger T. E. Delay E. R. (2009). Double P2X2/P2X3 purinergic receptor knockout mice do not taste NaCl or the artificial sweetener SC45647. Chem. Senses 34, 789–797 10.1093/chemse/bjp068 19833661 PMC2762055

[B65] El-Ajouz S. Ray D. Allsopp R. C. Evans R. J. (2012). Molecular basis of selective antagonism of the P2X1 receptor for ATP by NF449 and suramin: contribution of basic amino acids in the cysteine-rich loop. Br. J. Pharmacol. 165, 390–400 10.1111/j.1476-5381.2011.01534.x 21671897 PMC3268193

[B66] Ennion S. Hagan S. Evans R. J. (2000). The role of positively charged amino acids in ATP recognition by human P2X(1) receptors. J. Biol. Chem. 275, 29361–29367 10.1074/jbc.M003637200 10827197

[B67] Evans R. J. (2009). Orthosteric and allosteric binding sites of P2X receptors. Eur. Biophys. J. 38, 319–327 10.1007/s00249-008-0275-2 18247022

[B68] Evans R. J. (2010). Structural interpretation of P2X receptor mutagenesis studies on drug action. Br. J. Pharmacol. 161, 961–971 10.1111/j.1476-5381.2010.00728.x 20977449 PMC2972645

[B69] Finger T. E. Danilova V. Barrows J. Bartel D. L. Vigers A. J. Stone L. (2005). ATP signaling is crucial for communication from taste buds to gustatory nerves. Science 310, 1495–1499 10.1126/science.1118435 16322458

[B70] Fischer W. Zadori Z. Kullnick Y. Groger-Arndt H. Franke H. Wirkner K. (2007). Conserved lysin and arginin residues in the extracellular loop of P2X(3) receptors are involved in agonist binding. Eur. J. Pharmacol. 576, 7–17 10.1016/j.ejphar.2007.07.068 17764672

[B71] Franke H. Grosche J. Schadlich H. Krugel U. Allgaier C. Illes P. (2001). P2X receptor expression on astrocytes in the nucleus accumbens of rats. Neuroscience 108, 421–429 10.1016/S0306-4522(01)00416-X 11738256

[B72] Fujiwara Y. Kubo Y. (2004). Density-dependent changes of the pore properties of the P2X2 receptor channel. J. Physiol. 558, 31–43 10.1113/jphysiol.2004.064568 15107474 PMC1664918

[B73] Garcia-Guzman M. Soto F. Laube B. Stuhmer W. (1996). Molecular cloning and functional expression of a novel rat heart P2X purinoceptor. FEBS Lett. 388, 123–127 10.1016/0014-5793(96)00499-1 8690069

[B74] Gever J. R. Cockayne D. A. Dillon M. P. Burnstock G. Ford A. P. (2006). Pharmacology of P2X channels. Pflugers Arch. 452, 513–537 10.1007/s00424-006-0070-9 16649055

[B75] Glass R. Loesch A. Bodin P. Burnstock G. (2002). P2X4 and P2X6 receptors associate with VE-cadherin in human endothelial cells. Cell. Mol. Life Sci. 59, 870–881 10.1007/s00018-002-8474-y 12088286 PMC11146110

[B76] Gonzales E. B. Kawate T. Gouaux E. (2009). Pore architecture and ion sites in acid-sensing ion channels and P2X receptors. Nature 460, 599–604 10.1038/nature08218 19641589 PMC2845979

[B77] Groebe D. R. Dumm J. M. Levitan E. S. Abramson S. N. (1995). alpha-Conotoxins selectively inhibit one of the two acetylcholine binding sites of nicotinic receptors. Mol. Pharmacol. 48, 105–111 7623764

[B78] Grubb B. D. Evans R. J. (1999). Characterization of cultured dorsal root ganglion neuron P2X receptors. Eur. J. Neurosci. 11, 149–154 10.1046/j.1460-9568.1999.00426.x 9987019

[B79] Gu B. J. Zhang W. Worthington R. A. Sluyter R. Dao-Ung P. Petrou S. (2001). A Glu-496 to Ala polymorphism leads to loss of function of the human P2X7 receptor. J. Biol. Chem. 276, 11135–11142 10.1074/jbc.M010353200 11150303

[B80] Guo C. Masin M. Qureshi O. S. Murrell-Lagnado R. D. (2007). Evidence for functional P2X4/P2X7 heteromeric receptors. Mol. Pharmacol. 72, 1447–1456 10.1124/mol.107.035980 17785580

[B81] Haines W. R. Torres G. E. Voigt M. M. Egan T. M. (1999). Properties of the novel ATP-gated ionotropic receptor composed of the P2X(1) and P2X(5) isoforms. Mol. Pharmacol. 56, 720–727 10496954

[B82] Harhun M. I. Povstyan O. V. Gordienko D. V. (2010). Purinoreceptor-mediated current in myocytes from renal resistance arteries. Br. J. Pharmacol. 160, 987–997 10.1111/j.1476-5381.2010.00714.x 20590593 PMC2936003

[B83] Harrington L. S. Evans R. J. Wray J. Norling L. Swales K. E. Vial C. (2007). Purinergic 2X1 receptors mediate endothelial dependent vasodilation to ATP. Mol. Pharmacol. 72, 1132–1136 10.1124/mol.107.037325 17675587

[B84] Hattori M. Gouaux E. (2012). Molecular mechanism of ATP binding and ion channel activation in P2X receptors. Nature 485, 207–212 10.1038/nature11010 22535247 PMC3391165

[B85] Hausmann R. Bodnar M. Woltersdorf R. Wang H. Fuchs M. Messemer N. Illes P. (2012). ATP binding site mutagenesis reveals different subunit stoichiometry of functional P2X2/3 and P2X2/6 receptors. J. Biol. Chem. 287, 13930–13943 10.1074/jbc.M112.345207 22378790 PMC3340182

[B86] Hausmann R. Gunther J. Kless A. Kuhlmann D. Kassack M. U. Bahrenberg G. (2013). Salt bridge switching from Arg290/Glu167 to Arg290/ATP promotes the closed-to-open transition of the P2X2 receptor. Mol. Pharmacol. 83, 73–84 10.1124/mol.112.081489 23041661

[B87] Hernandez-Olmos V. Abdelrahman A. El-Tayeb A. Freudendahl D. Weinhausen S. Muller C. E. (2012). N-substituted phenoxazine and acridone derivatives: structure-activity relationships of potent P2X4 receptor antagonists. J. Med. Chem. 55, 9576–9588 10.1021/jm300845v 23075067

[B88] Hickman S. E. Kingery N. D. Ohsumi T. K. Borowsky M. L. Wang L. C. Means T. K. (2013). The microglial sensome revealed by direct RNA sequencing. Nat. Neurosci. 27, 3554 10.1038/nn.3554 24162652 PMC3840123

[B89] Jarvis M. F. (2010). The neural-glial purinergic receptor ensemble in chronic pain states. Trends Neurosci. 33, 48–57 10.1016/j.tins.2009.10.003 19914722

[B90] Jarvis M. F. Khakh B. S. (2009). ATP-gated P2X cation-channels. Neuropharmacology 56, 208–215 10.1016/j.neuropharm.2008.06.067 18657557

[B91] Jasti J. Furukawa H. Gonzales E. B. Gouaux E. (2007). Structure of acid-sensing ion channel 1 at 1.9[thinsp]A resolution and low pH. Nature 449, 316–323 10.1038/nature06163 17882215

[B92] Jensik P. J. Holbird D. Collard M. W. Cox T. C. (2001). Cloning and characterization of a functional P2X receptor from larval bullfrog skin. Am. J. Physiol. Cell Physiol. 281, C954–C962 11502572 10.1152/ajpcell.2001.281.3.C954

[B93] Jiang L. H. Kim M. Spelta V. Bo X. Surprenant A. North R. A. (2003). Subunit arrangement in P2X receptors. J. Neurosci. 23, 8903–8910 14523092 10.1523/JNEUROSCI.23-26-08903.2003 PMC6740386

[B94] Jiang L. H. Rassendren F. Surprenant A. North R. A. (2000). Identification of amino acid residues contributing to the ATP-binding site of a purinergic P2X receptor. J. Biol. Chem. 275, 34190–34196 10.1074/jbc.M005481200 10940304

[B95] Jiang R. Lemoine D. Martz A. Taly A. Gonin S. Prado De Carvalho L. (2011). Agonist trapped in ATP-binding sites of the P2X2 receptor. Proc. Natl. Acad. Sci. U.S.A. 108, 9066–9071 10.1073/pnas.1102170108 21576497 PMC3107266

[B96] Jiang R. Martz A. Gonin S. Taly A. De Carvalho L. P. Grutter T. (2010). A putative extracellular salt bridge at the subunit interface contributes to the ion channel function of the ATP-gated P2X2 receptor. J. Biol. Chem. 285, 15805–15815 10.1074/jbc.M110.101980 20308075 PMC2871448

[B97] Jiang R. Taly A. Lemoine D. Martz A. Cunrath O. Grutter T. (2012). Tightening of the ATP-binding sites induces the opening of P2X receptor channels. EMBO J. 31, 2134–2143 10.1038/emboj.2012.75 22473210 PMC3343472

[B98] Jones C. A. Chessell I. P. Simon J. Barnard E. A. Miller K. J. Michel A. D. (2000). Functional characterization of the P2X(4) receptor orthologues. Br. J. Pharmacol. 129, 388–394 10.1038/sj.bjp.0703059 10694247 PMC1571843

[B99] Jones C. A. Vial C. Sellers L. A. Humphrey P. P. Evans R. J. Chessell I. P. (2004). Functional regulation of P2X6 receptors by N-linked glycosylation: identification of a novel alpha beta-methylene ATP-sensitive phenotype. Mol. Pharmacol. 65, 979–985 10.1124/mol.65.4.979 15044628

[B100] Kaczmarek-Hajek K. Lorinczi E. Hausmann R. Nicke A. (2012). Molecular and functional properties of P2X receptors–recent progress and persisting challenges. Purinergic Signal. 8, 375–417 10.1007/s11302-012-9314-7 22547202 PMC3360091

[B101] Karoly R. Mike A. Illes P. Gerevich Z. (2008). The unusual state-dependent affinity of P2X3 receptors can be explained by an allosteric two-open-state model. Mol. Pharmacol. 73, 224–234 10.1124/mol.107.038901 17925459

[B102] Kawano A. Tsukimoto M. Mori D. Noguchi T. Harada H. Takenouchi T. (2012a). Regulation of P2X7-dependent inflammatory functions by P2X4 receptor in mouse macrophages. Biochem. Biophys. Res. Commun. 420, 102–107 10.1016/j.bbrc.2012.02.122 22405772

[B103] Kawano A. Tsukimoto M. Noguchi T. Hotta N. Harada H. Takenouchi T. (2012b). Involvement of P2X4 receptor in P2X7 receptor-dependent cell death of mouse macrophages. Biochem. Biophys. Res. Commun. 419, 374–380 10.1016/j.bbrc.2012.01.156 22349510

[B104] Kawate T. Michel J. C. Birdsong W. T. Gouaux E. (2009). Crystal structure of the ATP-gated P2X(4) ion channel in the closed state. Nature 460, 592–598 10.1038/nature08198 19641588 PMC2720809

[B105] Kawate T. Robertson J. L. Li M. Silberberg S. D. Swartz K. J. (2011). Ion access pathway to the transmembrane pore in P2X receptor channels. J. Gen. Physiol. 137, 579–590 10.1085/jgp.201010593 21624948 PMC3105519

[B106] Keceli B. Kubo Y. (2009). Functional and structural identification of amino acid residues of the P2X2 receptor channel critical for the voltage- and [ATP]-dependent gating. J. Physiol. 587, 5801–5818 10.1113/jphysiol.2009.182824 19884318 PMC2808541

[B107] Kennedy C. Tasker P. N. Gallacher G. Westfall T. D. (2007). Identification of atropine- and P2X1 receptor antagonist-resistant, neurogenic contractions of the urinary bladder. J. Neurosci. 27, 845–851 10.1523/JNEUROSCI.3115-06.2007 17251425 PMC6672915

[B108] Khadra A. Tomic M. Yan Z. Zemkova H. Sherman A. Stojilkovic S. S. (2013). Dual gating mechanism and function of P2X7 receptor channels. Biophys. J. 104, 2612–2621 10.1016/j.bpj.2013.05.006 23790369 PMC3686336

[B109] Khadra A. Yan Z. Coddou C. Tomic M. Sherman A. Stojilkovic S. S. (2012). Gating properties of the P2X2a and P2X2b receptor channels: experiments and mathematical modeling. J. Gen. Physiol. 139, 333–348 10.1085/jgp.201110716 22547664 PMC3343373

[B110] Khakh B. S. North R. A. (2012). Neuromodulation by extracellular ATP and P2X receptors in the CNS. Neuron 76, 51–69 10.1016/j.neuron.2012.09.024 23040806 PMC4064466

[B111] Khakh B. S. Proctor W. R. Dunwiddie T. V. Labarca C. Lester H. A. (1999). Allosteric control of gating and kinetics at P2X(4) receptor channels. J. Neurosci. 19, 7289–7299 10460235 10.1523/JNEUROSCI.19-17-07289.1999 PMC6782529

[B112] Khakh B. S. Smith W. B. Chiu C. S. Ju D. Davidson N. Lester H. A. (2001). Activation-dependent changes in receptor distribution and dendritic morphology in hippocampal neurons expressing P2X2-green fluorescent protein receptors. Proc. Natl. Acad. Sci. U.S.A. 98, 5288–5293 10.1073/pnas.081089198 11296257 PMC33202

[B113] King B. F. Liu M. Pintor J. Gualix J. Miras-Portugal M. T. Burnstock G. (1999). Diinosine pentaphosphate (IP5I) is a potent antagonist at recombinant rat P2X1 receptors. Br. J. Pharmacol. 128, 981–988 10.1038/sj.bjp.0702876 10556935 PMC1571720

[B114] King B. F. Townsend-Nicholson A. Wildman S. S. Thomas T. Spyer K. M. Burnstock G. (2000). Coexpression of rat P2X2 and P2X6 subunits in Xenopus oocytes. J. Neurosci. 20, 4871–4877 10864944 10.1523/JNEUROSCI.20-13-04871.2000 PMC6772291

[B115] King B. F. Wildman S. S. Ziganshina L. E. Pintor J. Burnstock G. (1997). Effects of extracellular pH on agonism and antagonism at a recombinant P2X2 receptor. Br. J. Pharmacol. 121, 1445–1453 10.1038/sj.bjp.0701286 9257926 PMC1564844

[B116] King B. F. Ziganshina L. E. Pintor J. Burnstock G. (1996). Full sensitivity of P2X2 purinoceptor to ATP revealed by changing extracellular pH. Br. J. Pharmacol. 117, 1371–1373 10.1111/j.1476-5381.1996.tb15293.x 8730726 PMC1909447

[B117] Klapperstück M. Büttner C. Schmalzing G. Markwardt F. (2001). Functional evidence of distinct ATP activation sites at the human P2X(7) receptor. J. Physiol. 534, 25–35 10.1111/j.1469-7793.2001.00025.x 11432989 PMC2278689

[B118] Kondratskaya E. Nonaka K. Akaike N. (2008). Influence of Purinergic Modulators on eEPSCs in Rat CA3 Hippocampal Neurons: Contribution of Ionotropic ATP Receptors. Neurophysiology 40, 21–29 10.1007/s11062-008-9011-x

[B119] Koshimizu T. A. Ueno S. Tanoue A. Yanagihara N. Stojilkovic S. S. Tsujimoto G. (2002). Heteromultimerization modulates P2X receptor functions through participating extracellular and C-terminal subdomains. J. Biol. Chem. 277, 46891–46899 10.1074/jbc.M205274200 12361958

[B120] Kukley M. Barden J. A. Steinhauser C. Jabs R. (2001). Distribution of P2X receptors on astrocytes in juvenile rat hippocampus. Glia 36, 11–21 10.1002/glia.1091 11571780

[B121] Lalo U. Pankratov Y. Wichert S. P. Rossner M. J. North R. A. Kirchhoff F. (2008). P2X1 and P2X5 subunits form the functional P2X receptor in mouse cortical astrocytes. J. Neurosci. 28, 5473–5480 10.1523/JNEUROSCI.1149-08.2008 18495881 PMC3844808

[B122] Lalo U. Verkhratsky A. Pankratov Y. (2007). Ivermectin potentiates ATP-induced ion currents in cortical neurones: evidence for functional expression of P2X4 receptors? Neurosci. Lett. 421, 158–162 10.1016/j.neulet.2007.03.078 17566648

[B123] Lalo U. V. Pankratov Y. V. Arndts D. Krishtal O. A. (2001). Omega-conotoxin GVIA potently inhibits the currents mediated by P2X receptors in rat DRG neurons. Brain Res. Bull. 54, 507–512 10.1016/S0361-9230(01)00433-6 11397540

[B124] Le K. T. Babinski K. Seguela P. (1998). Central P2X4 and P2X6 channel subunits coassemble into a novel heteromeric ATP receptor. J. Neurosci. 18, 7152–7159 9736638 10.1523/JNEUROSCI.18-18-07152.1998 PMC6793241

[B125] Le K. T. Boue-Grabot E. Archambault V. Seguela P. (1999). Functional and biochemical evidence for heteromeric ATP-gated channels composed of P2X1 and P2X5 subunits. J. Biol. Chem. 274, 15415–15419 10.1074/jbc.274.22.15415 10336430

[B126] Le K. T. Paquet M. Nouel D. Babinski K. Seguela P. (1997). Primary structure and expression of a naturally truncated human P2X ATP receptor subunit from brain and immune system. FEBS Lett. 418, 195–199 10.1016/S0014-5793(97)01380-X 9414125

[B127] Le Stunff H. Auger R. Kanellopoulos J. Raymond M. N. (2004). The Pro-451 to Leu polymorphism within the C-terminal tail of P2X7 receptor impairs cell death but not phospholipase D activation in murine thymocytes. J. Biol. Chem. 279, 16918–16926 10.1074/jbc.M313064200 14761980

[B128] Lewis C. J. Evans R. J. (2000). Comparison of P2X receptors in rat mesenteric, basilar and septal (coronary) arteries. J. Auton. Nerv. Syst. 81, 69–74 10.1016/S0165-1838(00)00117-X 10869703

[B129] Lewis C. J. Evans R. J. (2001). P2X receptor immunoreactivity in different arteries from the femoral, pulmonary, cerebral, coronary and renal circulations. J. Vasc. Res. 38, 332–340 10.1159/000051064 11455204

[B130] Lewis C. Neidhart S. Holy C. North R. A. Buell G. Surprenant A. (1995). Coexpression of P2X2 and P2X3 receptor subunits can account for ATP-gated currents in sensory neurons. Nature 377, 432–435 10.1038/377432a0 7566120

[B131] Li C. Peoples R. W. Weight F. F. (1996). Proton potentiation of ATP-gated ion channel responses to ATP and Zn2+ in rat nodose ganglion neurons. J. Neurophysiol. 76, 3048–3058 8930254 10.1152/jn.1996.76.5.3048

[B132] Li M. Silberberg S. D. Swartz K. J. (2013). Subtype-specific control of P2X receptor channel signaling by ATP and Mg2+. Proc. Natl. Acad. Sci. U.S.A. 110, E3455–E3463 10.1073/pnas.1308088110 23959888 PMC3767550

[B133] Liang L. Zsembery A. Schwiebert E. M. (2005). RNA interference targeted to multiple P2X receptor subtypes attenuates zinc-induced calcium entry. Am. J. Physiol. Cell Physiol. 289, C388–C396 10.1152/ajpcell.00491.2004 15800050

[B134] Liu M. King B. F. Dunn P. M. Rong W. Townsend-Nicholson A. Burnstock G. (2001). Coexpression of P2X(3) and P2X(2) receptor subunits in varying amounts generates heterogeneous populations of P2X receptors that evoke a spectrum of agonist responses comparable to that seen in sensory neurons. J. Pharmacol. Exp. Ther. 296, 1043–1050 11181939

[B135] Lörinczi E. Bhargava Y. Marino S. F. Taly A. Kaczmarek-Hajek K. Barrantes-Freer A. (2012). Involvement of the cysteine-rich head domain in activation and desensitization of the P2X1 receptor. Proc. Natl. Acad. Sci. U.S.A. 109, 11396–11401 10.1073/pnas.1118759109 22745172 PMC3396496

[B136] Ma W. Korngreen A. Weil S. Cohen E. B. Priel A. Kuzin L. (2006). Pore properties and pharmacological features of the P2X receptor channel in airway ciliated cells. J. Physiol. 571, 503–517 10.1113/jphysiol.2005.103408 16423852 PMC1805806

[B137] Maria G. N. Raquel G. A. Eduardo V. M. Rosa E. L. Nestor J. V. Andromeda L. R. (2013). P2X4 subunits are part of P2X native channels in murine myenteric neurons. Eur. J. Pharmacol. 709, 93–102 10.1016/j.ejphar.2013.03.045 23567069

[B138] Marquez-Klaka B. Rettinger J. Bhargava Y. Eisele T. Nicke A. (2007). Identification of an intersubunit cross-link between substituted cysteine residues located in the putative ATP binding site of the P2X1 receptor. J. Neurosci. 27, 1456–1466 10.1523/JNEUROSCI.3105-06.2007 17287520 PMC6673578

[B139] Marquez-Klaka B. Rettinger J. Nicke A. (2009). Inter-subunit disulfide cross-linking in homomeric and heteromeric P2X receptors. Eur. Biophys. J. 38, 329–338 10.1007/s00249-008-0325-9 18427801

[B140] Matute C. Torre I. Perez-Cerda F. Perez-Samartin A. Alberdi E. Etxebarria E. (2007). P2X(7) receptor blockade prevents ATP excitotoxicity in oligodendrocytes and ameliorates experimental autoimmune encephalomyelitis. J. Neurosci. 27, 9525–9533 10.1523/JNEUROSCI.0579-07.2007 17728465 PMC6673129

[B141] Michel A. D. Grahames C. B. Humphrey P. P. (1996). Functional characterisation of P2 purinoceptors in PC12 cells by measurement of radiolabelled calcium influx. Naunyn Schmiedebergs. Arch. Pharmacol. 354, 562–571 10.1007/BF00170829 8938653

[B142] Mio K. Kubo Y. Ogura T. Yamamoto T. Sato C. (2005). Visualization of the trimeric P2X2 receptor with a crown-capped extracellular domain. Biochem. Biophys. Res. Commun. 337, 998–1005 10.1016/j.bbrc.2005.09.141 16219297

[B143] Mori M. Heuss C. Gahwiler B. H. Gerber U. (2001). Fast synaptic transmission mediated by P2X receptors in CA3 pyramidal cells of rat hippocampal slice cultures. J. Physiol. 535, 115–123 10.1111/j.1469-7793.2001.t01-1-00115.x 11507162 PMC2278762

[B144] Nagaya N. Tittle R. K. Saar N. Dellal S. S. Hume R. I. (2005). An intersubunit zinc binding site in rat P2X2 receptors. J. Biol. Chem. 280, 25982–25993 10.1074/jbc.M504545200 15899882 PMC1479454

[B145] Nawa G. Urano T. Tokino T. Ochi T. Miyoshi Y. (1998). Cloning and characterization of the murine P2XM receptor gene. J. Hum. Genet. 43, 262–267 10.1007/s100380050086 9852680

[B146] Nicke A. (2008). Homotrimeric complexes are the dominant assembly state of native P2X7 subunits. Biochem. Biophys. Res. Commun. 377, 803–808 10.1016/j.bbrc.2008.10.042 18938136

[B147] Nicke A. Baumert H. G. Rettinger J. Eichele A. Lambrecht G. Mutschler E. (1998). P2X1 and P2X3 receptors form stable trimers: a novel structural motif of ligand-gated ion channels. EMBO J. 17, 3016–3028 10.1093/emboj/17.11.3016 9606184 PMC1170641

[B148] Nicke A. Kerschensteiner D. Soto F. (2005). Biochemical and functional evidence for heteromeric assembly of P2X1 and P2X4 subunits. J. Neurochem. 92, 925–933 10.1111/j.1471-4159.2004.02939.x 15686495

[B149] Nörenberg W. Schunk J. Fischer W. Sobottka H. Riedel T. Oliveira J. F. (2010). Electrophysiological classification of P2X7 receptors in rat cultured neocortical astroglia. Br. J. Pharmacol. 160, 1941–1952 10.1111/j.1476-5381.2010.00736.x 20649592 PMC2958640

[B150] Nori S. Fumagalli L. Bo X. Bogdanov Y. Burnstock G. (1998). Coexpression of mRNAs for P2X1, P2X2 and P2X4 receptors in rat vascular smooth muscle: an *in situ* hybridization and RT-PCR study. J. Vasc. Res. 35, 179–185 10.1159/000025582 9647332

[B151] North R. A. (2002). Molecular physiology of P2X receptors. Physiol. Rev. 82, 1013–1067 12270951 10.1152/physrev.00015.2002

[B152] Ormond S. J. Barrera N. P. Qureshi O. S. Henderson R. M. Edwardson J. M. Murrell-Lagnado R. D. (2006). An uncharged region within the N terminus of the P2X6 receptor inhibits its assembly and exit from the endoplasmic reticulum. Mol. Pharmacol. 69, 1692–1700 10.1124/mol.105.020404 16452399

[B153] Patel M. K. Khakh B. S. Henderson G. (2001). Properties of native P2X receptors in rat trigeminal mesencephalic nucleus neurones: lack of correlation with known, heterologously expressed P2X receptors. Neuropharmacology 40, 96–105 10.1016/S0028-3908(00)00108-8 11077075

[B154] Petruska J. C. Napaporn J. Johnson R. D. Cooper B. Y. (2002). Chemical responsiveness and histochemical phenotype of electrophysiologically classified cells of the adult rat dorsal root ganglion. Neuroscience 115, 15–30 10.1016/S0306-4522(02)00409-8 12401318

[B155] Phillips J. K. Hill C. E. (1999). Neuroreceptor mRNA expression in the rat mesenteric artery develops independently of innervation. Int. J. Dev. Neurosci. 17, 377–386 10.1016/S0736-5748(99)00032-5 10479072

[B156] Pintor J. King B. F. Miras-Portugal M. T. Burnstock G. (1996). Selectivity and activity of adenine dinucleotides at recombinant P2X2 and P2Y1 purinoceptors. Br. J. Pharmacol. 119, 1006–1012 10.1111/j.1476-5381.1996.tb15771.x 8922753 PMC1915941

[B157] Priel A. Silberberg S. D. (2004). Mechanism of ivermectin facilitation of human P2X4 receptor channels. J. Gen. Physiol. 123, 281–293 10.1085/jgp.200308986 14769846 PMC2217454

[B158] Qureshi O. S. Paramasivam A. Yu J. C. Murrell-Lagnado R. D. (2007). Regulation of P2X4 receptors by lysosomal targeting, glycan protection and exocytosis. J. Cell Sci. 120, 3838–3849 10.1242/jcs.010348 17940064

[B159] Radford K. M. Virginio C. Surprenant A. North R. A. Kawashima E. (1997). Baculovirus expression provides direct evidence for heteromeric assembly of P2X2 and P2X3 receptors. J. Neurosci. 17, 6529–6533 9254665 10.1523/JNEUROSCI.17-17-06529.1997 PMC6573133

[B160] Raouf R. Chabot-Dore A. J. Ase A. R. Blais D. Seguela P. (2007). Differential regulation of microglial P2X4 and P2X7 ATP receptors following LPS-induced activation. Neuropharmacology 53, 496–504 10.1016/j.neuropharm.2007.06.010 17675190

[B161] Rassendren F. Buell G. N. Virginio C. Collo G. North R. A. Surprenant A. (1997). The permeabilizing ATP receptor, P2X7. Cloning and expression of a human cDNA. J. Biol. Chem. 272, 5482–5486 10.1074/jbc.272.9.5482 9038151

[B162] Resende R. R. Britto L. R. Ulrich H. (2008). Pharmacological properties of purinergic receptors and their effects on proliferation and induction of neuronal differentiation of P19 embryonal carcinoma cells. Int. J. Dev. Neurosci. 26, 763–777 10.1016/j.ijdevneu.2008.07.008 18675894

[B163] Resende R. R. Majumder P. Gomes K. N. Britto L. R. Ulrich H. (2007). P19 embryonal carcinoma cells as *in vitro* model for studying purinergic receptor expression and modulation of N-methyl-D-aspartate-glutamate and acetylcholine receptors during neuronal differentiation. Neuroscience 146, 1169–1181 10.1016/j.neuroscience.2007.02.041 17418494

[B164] Rettinger J. Braun K. Hochmann H. Kassack M. U. Ullmann H. Nickel P. (2005). Profiling at recombinant homomeric and heteromeric rat P2X receptors identifies the suramin analogue NF449 as a highly potent P2X1 receptor antagonist. Neuropharmacology 48, 461–468 10.1016/j.neuropharm.2004.11.003 15721178

[B165] Rettinger J. Schmalzing G. Damer S. Muller G. Nickel P. Lambrecht G. (2000). The suramin analogue NF279 is a novel and potent antagonist selective for the P2X(1) receptor. Neuropharmacology 39, 2044–2053 10.1016/S0028-3908(00)00022-8 10963748

[B166] Roberts J. A. Allsopp R. C. El Ajouz S. Vial C. Schmid R. Young M. T. (2012). Agonist binding evokes extensive conformational changes in the extracellular domain of the ATP-gated human P2X1 receptor ion channel. Proc. Natl. Acad. Sci. U.S.A. 109, 4663–4667 10.1073/pnas.1201872109 22393010 PMC3311380

[B167] Roberts J. A. Digby H. R. Kara M. El Ajouz S. Sutcliffe M. J. Evans R. J. (2008). Cysteine substitution mutagenesis and the effects of methanethiosulfonate reagents at P2X2 and P2X4 receptors support a core common mode of ATP action at P2X receptors. J. Biol. Chem. 283, 20126–20136 10.1074/jbc.M800294200 18487206 PMC2459275

[B168] Roberts J. A. Evans R. J. (2004). ATP binding at human P2X1 receptors. Contribution of aromatic and basic amino acids revealed using mutagenesis and partial agonists. J. Biol. Chem. 279, 9043–9055 10.1074/jbc.M308964200 14699168

[B169] Roberts J. A. Evans R. J. (2006). Contribution of conserved polar glutamine, asparagine and threonine residues and glycosylation to agonist action at human P2X1 receptors for ATP. J. Neurochem. 96, 843–852 10.1111/j.1471-4159.2005.03593.x 16371009

[B170] Roberts J. A. Valente M. Allsopp R. C. Watt D. Evans R. J. (2009). Contribution of the region Glu181 to Val200 of the extracellular loop of the human P2X1 receptor to agonist binding and gating revealed using cysteine scanning mutagenesis. J. Neurochem. 109, 1042–1052 10.1111/j.1471-4159.2009.06035.x 19519776 PMC2695859

[B171] Roger S. Mei Z. Z. Baldwin J. M. Dong L. Bradley H. Baldwin S. A. (2010). Single nucleotide polymorphisms that were identified in affective mood disorders affect ATP-activated P2X7 receptor functions. J. Psychiatr. Res. 44, 347–355 10.1016/j.jpsychires.2009.10.005 19931869

[B172] Rokic M. B. Tvrdonova V. Vavra V. Jindrichova M. Obsil T. Stojilkovic S. S. (2010). Roles of conserved ectodomain cysteines of the rat P2X4 purinoreceptor in agonist binding and channel gating. Physiol. Res. 59, 927–935 20406028 10.33549/physiolres.931979 PMC3170524

[B173] Ross F. M. Brodie M. J. Stone T. W. (1998). Modulation by adenine nucleotides of epileptiform activity in the CA3 region of rat hippocampal slices. Br. J. Pharmacol. 123, 71–80 10.1038/sj.bjp.0701586 9484856 PMC1565143

[B174] Rubio M. E. Soto F. (2001). Distinct Localization of P2X receptors at excitatory postsynaptic specializations. J. Neurosci. 21, 641–653 11160443 10.1523/JNEUROSCI.21-02-00641.2001 PMC6763822

[B175] Ruppelt A. Ma W. Borchardt K. Silberberg S. D. Soto F. (2001). Genomic structure, developmental distribution and functional properties of the chicken P2X(5) receptor. J. Neurochem. 77, 1256–1265 10.1046/j.1471-4159.2001.00348.x 11389176

[B176] Sakaki H. Fujiwaki T. Tsukimoto M. Kawano A. Harada H. Kojima S. (2013). P2X4 receptor regulates P2X7 receptor-dependent IL-1beta and IL-18 release in mouse bone marrow-derived dendritic cells. Biochem. Biophys. Res. Commun. 432, 406–411 10.1016/j.bbrc.2013.01.135 23428419

[B177] Samways D. S. Khakh B. S. Dutertre S. Egan T. M. (2011). Preferential use of unobstructed lateral portals as the access route to the pore of human ATP-gated ion channels (P2X receptors). Proc. Natl. Acad. Sci. U.S.A. 108, 13800–13805 10.1073/pnas.1017550108 21808018 PMC3158179

[B178] Schwarz N. Drouot L. Nicke A. Fliegert R. Boyer O. Guse A. H. (2012). Alternative splicing of the N-terminal cytosolic and transmembrane domains of P2X7 controls gating of the ion channel by ADP-ribosylation. PLoS ONE 7:e41269 10.1371/journal.pone.0041269 22848454 PMC3407210

[B179] Schwindt T. T. Trujillo C. A. Negraes P. D. Lameu C. Ulrich H. (2011). Directed differentiation of neural progenitors into neurons is accompanied by altered expression of P2X purinergic receptors. J. Mol. Neurosci. 44, 141–146 10.1007/s12031-010-9417-y 20617399

[B180] Serrano A. Mo G. Grant R. Pare M. O'Donnell D. Yu X. H. (2012). Differential expression and pharmacology of native P2X receptors in rat and primate sensory neurons. J. Neurosci. 32, 11890–11896 10.1523/JNEUROSCI.0698-12.2012 22915129 PMC6703778

[B181] Sim J. A. Chaumont S. Jo J. Ulmann L. Young M. T. Cho K. (2006). Altered hippocampal synaptic potentiation in P2X4 knock-out mice. J. Neurosci. 26, 9006–9009 10.1523/JNEUROSCI.2370-06.2006 16943557 PMC6675341

[B182] Sim J. A. Park C. K. Oh S. B. Evans R. J. North R. A. (2007). P2X1 and P2X4 receptor currents in mouse macrophages. Br. J. Pharmacol. 152, 1283–1290 10.1038/sj.bjp.0707504 17934511 PMC2034508

[B183] Skals M. Jorgensen N. R. Leipziger J. Praetorius H. A. (2009). Alpha-hemolysin from Escherichia coli uses endogenous amplification through P2X receptor activation to induce hemolysis. Proc. Natl. Acad. Sci. U.S.A. 106, 4030–4035 10.1073/pnas.0807044106 19225107 PMC2656199

[B184] Sluyter R. Barden J. A. Wiley J. S. (2001). Detection of P2X purinergic receptors on human B lymphocytes. Cell Tissue Res. 304, 231–236 10.1007/s004410100372 11396717

[B185] Sorge R. E. Trang T. Dorfman R. Smith S. B. Beggs S. Ritchie J. (2012). Genetically determined P2X7 receptor pore formation regulates variability in chronic pain sensitivity. Nat. Med. 18, 595–599 10.1038/nm.2710 22447075 PMC3350463

[B186] Soto F. Garcia-Guzman M. Gomez-Hernandez J. M. Hollmann M. Karschin C. Stuhmer W. (1996a). P2X4: an ATP-activated ionotropic receptor cloned from rat brain. Proc. Natl. Acad. Sci. U.S.A. 93, 3684–3688 10.1073/pnas.93.8.3684 8622997 PMC39672

[B187] Soto F. Garcia-Guzman M. Karschin C. Stuhmer W. (1996b). Cloning and tissue distribution of a novel P2X receptor from rat brain. Biochem. Biophys. Res. Commun. 223, 456–460 10.1006/bbrc.1996.0915 8670303

[B188] Spelta V. Jiang L. H. Surprenant A. North R. A. (2002). Kinetics of antagonist actions at rat P2X2/3 heteromeric receptors. Br. J. Pharmacol. 135, 1524–1530 10.1038/sj.bjp.0704591 11906966 PMC1573256

[B189] Spelta V. Mekhalfia A. Rejman D. Thompson M. Blackburn G. M. North R. A. (2003). ATP analogues with modified phosphate chains and their selectivity for rat P2X2 and P2X2/3 receptors. Br. J. Pharmacol. 140, 1027–1034 10.1038/sj.bjp.0705531 14581175 PMC1574118

[B190] Stelmashenko O. Lalo U. Yang Y. Bragg L. North R. A. Compan V. (2012). Activation of trimeric P2X2 receptors by fewer than three ATP molecules. Mol. Pharmacol. 82, 760–766 10.1124/mol.112.080903 22828800 PMC3463222

[B191] Stokes L. Surprenant A. (2009). Dynamic regulation of the P2X4 receptor in alveolar macrophages by phagocytosis and classical activation. Eur. J. Immunol. 39, 986–995 10.1002/eji.200838818 19283779

[B192] Stoop R. Surprenant A. North R. A. (1997). Different sensitivities to pH of ATP-induced currents at four cloned P2X receptors. J. Neurophysiol. 78, 1837–1840 9325352 10.1152/jn.1997.78.4.1837

[B193] Sun J. H. Cai G. J. Xiang Z. H. (2007). Expression of P2X purinoceptors in PC12 phaeochromocytoma cells. Clin. Exp. Pharmacol. Physiol. 34, 1282–1286 10.1111/j.1440-1681.2007.04718.x 17973868

[B194] Surprenant A. North R. A. (2009). Signaling at purinergic P2X receptors. Annu. Rev. Physiol. 71, 333–359 10.1146/annurev.physiol.70.113006.100630 18851707

[B195] Surprenant A. Schneider D. A. Wilson H. L. Galligan J. J. North R. A. (2000). Functional properties of heteromeric P2X(1/5) receptors expressed in HEK cells and excitatory junction potentials in guinea-pig submucosal arterioles. J. Auton. Nerv. Syst. 81, 249–263 10.1016/S0165-1838(00)00123-5 10869729

[B196] Thomas S. Virginio C. North R. A. Surprenant A. (1998). The antagonist trinitrophenyl-ATP reveals co-existence of distinct P2X receptor channels in rat nodose neurones. J. Physiol. 509(Pt 2), 411–417 10.1111/j.1469-7793.1998.411bn.x 9575290 PMC2230974

[B197] Tittle R. K. Hume R. I. (2008). Opposite effects of zinc on human and rat P2X2 receptors. J. Neurosci. 28, 11131–11140 10.1523/JNEUROSCI.2763-08.2008 18971456 PMC2586956

[B198] Torres G. E. Egan T. M. Voigt M. M. (1999a). Hetero-oligomeric assembly of P2X receptor subunits. Specificities exist with regard to possible partners. J. Biol. Chem. 274, 6653–6659 10.1074/jbc.274.10.6653 10037762

[B199] Torres G. E. Egan T. M. Voigt M. M. (1999b). Identification of a domain involved in ATP-gated ionotropic receptor subunit assembly. J. Biol. Chem. 274, 22359–22365 10.1074/jbc.274.32.22359 10428806

[B200] Torres G. E. Haines W. R. Egan T. M. Voigt M. M. (1998). Co-expression of P2X1 and P2X5 receptor subunits reveals a novel ATP-gated ion channel. Mol. Pharmacol. 54, 989–993 9855626 10.1124/mol.54.6.989

[B201] Toulme E. Garcia A. Samways D. Egan T. M. Carson M. J. Khakh B. S. (2010). P2X4 receptors in activated C8-B4 cells of cerebellar microglial origin. J. Gen. Physiol. 135, 333–353 10.1085/jgp.200910336 20231374 PMC2847917

[B202] Tsuda M. Shigemoto-Mogami Y. Koizumi S. Mizokoshi A. Kohsaka S. Salter M. W. (2003). P2X4 receptors induced in spinal microglia gate tactile allodynia after nerve injury. Nature 424, 778–783 10.1038/nature01786 12917686

[B203] Ueno S. Tsuda M. Iwanaga T. Inoue K. (1999). Cell type-specific ATP-activated responses in rat dorsal root ganglion neurons. Br. J. Pharmacol. 126, 429–436 10.1038/sj.bjp.0702319 10077235 PMC1565824

[B204] Ulmann L. Hatcher J. P. Hughes J. P. Chaumont S. Green P. J. Conquet F. (2008). Up-regulation of P2X4 receptors in spinal microglia after peripheral nerve injury mediates BDNF release and neuropathic pain. J. Neurosci. 28, 11263–11268 10.1523/JNEUROSCI.2308-08.2008 18971468 PMC6671487

[B205] Ulmann L. Hirbec H. Rassendren F. (2010). P2X4 receptors mediate PGE2 release by tissue-resident macrophages and initiate inflammatory pain. EMBO J. 29, 2290–2300 10.1038/emboj.2010.126 20562826 PMC2910276

[B206] Virginio C. Church D. North R. A. Surprenant A. (1997). Effects of divalent cations, protons and calmidazolium at the rat P2X7 receptor. Neuropharmacology 36, 1285–1294 10.1016/S0028-3908(97)00141-X 9364483

[B207] Virginio C. Mackenzie A. North R. A. Surprenant A. (1999a). Kinetics of cell lysis, dye uptake and permeability changes in cells expressing the rat P2X7 receptor. J. Physiol. 519(Pt 2), 335–346 10.1111/j.1469-7793.1999.0335m.x 10457053 PMC2269518

[B208] Virginio C. Mackenzie A. Rassendren F. A. North R. A. Surprenant A. (1999b). Pore dilation of neuronal P2X receptor channels. Nat. Neurosci. 2, 315–321 10.1038/7225 10204537

[B209] Virginio C. Robertson G. Surprenant A. North R. A. (1998). Trinitrophenyl-substituted nucleotides are potent antagonists selective for P2X1, P2X3, and heteromeric P2X2/3 receptors. Mol. Pharmacol. 53, 969–973 9614197

[B210] Vlaskovska M. Kasakov L. Rong W. Bodin P. Bardini M. Cockayne D. A. (2001). P2X3 knock-out mice reveal a major sensory role for urothelially released ATP. J. Neurosci. 21, 5670–5677 11466438 10.1523/JNEUROSCI.21-15-05670.2001 PMC6762653

[B211] Wang L. Jacobsen S. E. Bengtsson A. Erlinge D. (2004). P2 receptor mRNA expression profiles in human lymphocytes, monocytes and CD34+ stem and progenitor cells. BMC Immunol. 5:16 10.1186/1471-2172-5-16 15291969 PMC509419

[B212] Wareham K. Vial C. Wykes R. C. Bradding P. Seward E. P. (2009). Functional evidence for the expression of P2X1, P2X4 and P2X7 receptors in human lung mast cells. Br. J. Pharmacol. 157, 1215–1224 10.1111/j.1476-5381.2009.00287.x 19552691 PMC2743840

[B213] Weinhold K. Krause-Buchholz U. Rodel G. Kasper M. Barth K. (2010). Interaction and interrelation of P2X7 and P2X4 receptor complexes in mouse lung epithelial cells. Cell. Mol. Life Sci. 67, 2631–2642 10.1007/s00018-010-0355-1 20405163 PMC11115700

[B214] Welter-Stahl L. Da Silva C. M. Schachter J. Persechini P. M. Souza H. S. Ojcius D. M. (2009). Expression of purinergic receptors and modulation of P2X7 function by the inflammatory cytokine IFNgamma in human epithelial cells. Biochim. Biophys. Acta 1788, 1176–1187 10.1016/j.bbamem.2009.03.006 19306841

[B215] Wildman S. S. Brown S. G. King B. F. Burnstock G. (1999a). Selectivity of diadenosine polyphosphates for rat P2X receptor subunits. Eur. J. Pharmacol. 367, 119–123 10.1016/S0014-2999(98)00976-5 10082274

[B216] Wildman S. S. King B. F. Burnstock G. (1999b). Modulation of ATP-responses at recombinant rP2X4 receptors by extracellular pH and zinc. Br. J. Pharmacol. 126, 762–768 10.1038/sj.bjp.0702325 10188989 PMC1565836

[B217] Wildman S. S. Brown S. G. Rahman M. Noel C. A. Churchill L. Burnstock G. (2002). Sensitization by extracellular Ca(2+) of rat P2X(5) receptor and its pharmacological properties compared with rat P2X(1). Mol. Pharmacol. 62, 957–966 10.1124/mol.62.4.957 12237343

[B218] Wilkinson W. J. Jiang L. H. Surprenant A. North R. A. (2006). Role of ectodomain lysines in the subunits of the heteromeric P2X2/3 receptor. Mol. Pharmacol. 70, 1159–1163 10.1124/mol.106.026658 16840712

[B219] Wilson H. L. Varcoe R. W. Stokes L. Holland K. L. Francis S. E. Dower S. K. (2007). P2X receptor characterization and IL-1/IL-1Ra release from human endothelial cells. Br. J. Pharmacol. 151, 115–127 10.1038/sj.bjp.0707213 17351655 PMC2012976

[B220] Wirkner K. Sperlagh B. Illes P. (2007). P2X3 receptor involvement in pain states. Mol. Neurobiol. 36, 165–183 10.1007/s12035-007-0033-y 17952660

[B221] Woehrle T. Yip L. Elkhal A. Sumi Y. Chen Y. Yao Y. (2010a). Pannexin-1 hemichannel-mediated ATP release together with P2X1 and P2X4 receptors regulate T-cell activation at the immune synapse. Blood 116, 3475–3484 10.1182/blood-2010-04-277707 20660288 PMC2981474

[B222] Woehrle T. Yip L. Manohar M. Sumi Y. Yao Y. Chen Y. (2010b). Hypertonic stress regulates T cell function via pannexin-1 hemichannels and P2X receptors. J. Leukoc. Biol. 88, 1181–1189 10.1189/jlb.0410211 20884646 PMC2996895

[B223] Wolf C. Rosefort C. Fallah G. Kassack M. U. Hamacher A. Bodnar M. (2011). Molecular determinants of potent P2X2 antagonism identified by functional analysis, mutagenesis, and homology docking. Mol. Pharmacol. 79, 649–661 10.1124/mol.110.068700 21191044

[B224] Worthington R. A. Dutton J. L. Poronnik P. Bennett M. R. Barden J. A. (1999). Localisation of P2X receptors in human salivary gland epithelial cells and human embryonic kidney cells by sodium dodecyl sulfate-polyacrylamide gel electrophoresis/Western blotting and immunofluorescence. Electrophoresis 20, 2065–2070 10.1002/(SICI)1522-2683(19990701)20:10<2065::AID-ELPS2065>3.0.CO;2-E 10451116

[B225] Xiang Z. Bo X. Burnstock G. (1998). Localization of ATP-gated P2X receptor immunoreactivity in rat sensory and sympathetic ganglia. Neurosci. Lett. 256, 105–108 10.1016/S0304-3940(98)00774-5 9853714

[B226] Xu X. J. Boumechache M. Robinson L. E. Marschall V. Gorecki D. C. Masin M. (2012). Splice variants of the P2X7 receptor reveal differential agonist dependence and functional coupling with pannexin-1. J. Cell Sci. 125, 3776–3789 10.1242/jcs.099374 22553206

[B227] Yamamoto K. Korenaga R. Kamiya A. Qi Z. Sokabe M. Ando J. (2000). P2X(4) receptors mediate ATP-induced calcium influx in human vascular endothelial cells. Am. J. Physiol. Heart Circ. Physiol. 279, H285–H292 10899068 10.1152/ajpheart.2000.279.1.H285

[B228] Yamamoto M. Kamatsuka Y. Ohishi A. Nishida K. Nagasawa K. (2013). P2X7 receptors regulate engulfing activity of non-stimulated resting astrocytes. Biochem. Biophys. Res. Commun. 2013, 022 10.1016/j.bbrc.2013.08.022 23958305

[B229] Yan Z. Khadra A. Li S. Tomic M. Sherman A. Stojilkovic S. S. (2010). Experimental characterization and mathematical modeling of P2X7 receptor channel gating. J. Neurosci. 30, 14213–14224 10.1523/JNEUROSCI.2390-10.2010 20962242 PMC2980950

[B230] Yan Z. Khadra A. Sherman A. Stojilkovic S. S. (2011). Calcium-dependent block of P2X7 receptor channel function is allosteric. J. Gen. Physiol. 138, 437–452 10.1085/jgp.201110647 21911484 PMC3182445

[B231] Yan Z. Liang Z. Tomic M. Obsil T. Stojilkovic S. S. (2005). Molecular determinants of the agonist binding domain of a P2X receptor channel. Mol. Pharmacol. 67, 1078–1088 10.1124/mol.104.010108 15632318

[B232] Young M. T. Fisher J. A. Fountain S. J. Ford R. C. North R. A. Khakh B. S. (2008). Molecular shape, architecture, and size of P2X4 receptors determined using fluorescence resonance energy transfer and electron microscopy. J. Biol. Chem. 283, 26241–26251 10.1074/jbc.M804458200 18635539 PMC2533801

[B233] Young M. T. Pelegrin P. Surprenant A. (2006). Identification of Thr283 as a key determinant of P2X7 receptor function. Br. J. Pharmacol. 149, 261–268 10.1038/sj.bjp.0706880 16940988 PMC2014266

[B234] Yuahasi K. K. Demasi M. A. Tamajusuku A. S. Lenz G. Sogayar M. C. Fornazari M. (2012). Regulation of neurogenesis and gliogenesis of retinoic acid-induced P19 embryonal carcinoma cells by P2X2 and P2X7 receptors studied by RNA interference. Int. J. Dev. Neurosci. 30, 91–97 10.1016/j.ijdevneu.2011.12.010 22248690

[B235] Zemkova H. Yan Z. Liang Z. Jelinkova I. Tomic M. Stojilkovic S. S. (2007). Role of aromatic and charged ectodomain residues in the P2X(4) receptor functions. J. Neurochem. 102, 1139–1150 10.1111/j.1471-4159.2007.04616.x 17663752

[B236] Zhong Y. Banning A. S. Cockayne D. A. Ford A. P. Burnstock G. McMahon S. B. (2003). Bladder and cutaneous sensory neurons of the rat express different functional P2X receptors. Neuroscience 120, 667–675 10.1016/S0306-4522(03)00243-4 12895508

[B237] Zhong Y. Dunn P. M. Bardini M. Ford A. P. Cockayne D. A. Burnstock G. (2001). Changes in P2X receptor responses of sensory neurons from P2X3-deficient mice. Eur. J. Neurosci. 14, 1784–1792 10.1046/j.0953-816x.2001.01805.x 11860473

